# Embedding alkenes within an icosahedral inorganic fullerene {(NH_4_)_42_[Mo_132_O_372_(L)_30_(H_2_O)_72_]} for trapping volatile organics†

**DOI:** 10.1039/c9sc06217c

**Published:** 2020-01-23

**Authors:** Robert W. Pow, Weimin Xuan, De-Liang Long, Nicola L. Bell, Leroy Cronin

**Affiliations:** School of Chemistry, University of Glasgow University Avenue Glasgow G12 8QQ UK Lee.Cronin@Glasgow.ac.uk

## Abstract

Eight alkene-functionalized molybdenum-based spherical Keplerate-type (inorganic fullerene) structures have been obtained *via* both direct and multistep synthetic approaches. Driven by the opportunity to design unique host–guest interactions within hydrophobic, π-electron rich confined environments, we have synthesised {(NH_4_)_42_[Mo_132_O_372_(L)_30_(H_2_O)_72_]}, where L = (**1**) acrylic acid, (**2**) crotonic acid, (**3**) methacrylic acid, (**4**) tiglic acid, (**5**) 3-butenoic acid, (**6**) 4-pentenoic acid, (**7**) 5-hexenoic acid, and (**8**) sorbic acid. The compounds, which are obtained in good yield (10–40%), contain 30 carboxylate-coordinated alkene ligands which create a central cavity with hydrophobic character. Extensive Nuclear Magnetic Resonance (NMR) spectroscopy studies contribute significantly to the complete characterisation of the structures obtained, including both 1D and 2D measurements. In addition, single-crystal X-ray crystallography and subsequently-generated electron density maps are employed to highlight the distribution in ligand tail positions. These alkene-containing structures are shown to effectively encapsulate small alkyl thiols (1-propanethiol (**A**), 2-propanethiol (**B**), 1-butanethiol (**C**), 2-butanethiol (**D**) and 2-methyl-1-propanethiol (**E**)) as guests within the central cavity in aqueous solution. The hydrophobically driven clustering of up to 6 equivalents of volatile thiol guests within the central cavity of the Keplerate-type structure results in effective thermal protection, preventing evaporation at elevated temperatures (Δ*T* ≈ 25 K).

## Abstract 2

Eight alkene-functionalized molybdenum-based spherical Keplerate-type (inorganic fullerene) structures have been obtained and shown to sequester and thermally insulate volatile organics.

## Graphical abstract

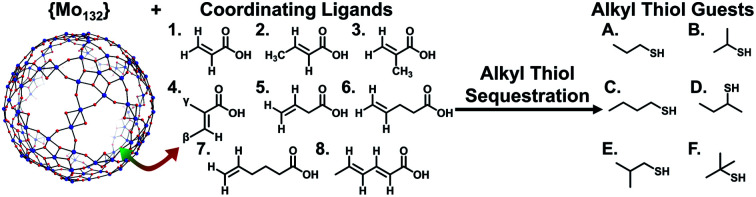

## Introduction

Host–guest encapsulation processes, facilitated by non-covalent interactions, are utilized in drug delivery,^1^ sensing,^2^ and separation processes.^3^ Inorganic systems have provided insights into the driving force for host–guest interactions such as the hydrophobically driven uptake of alcohols^4^ and the enthalpically dominated encapsulation of organic guests within a M_8_L_12_ metal/ligand coordination cage.^5^ Polyoxometalates (POMs) are discrete metal-oxide clusters which exhibit varied architectures, sizes and compositions. High-nuclearity, spherical Keplerate-type POMs,^6^ have been used as hosts in previous guest encapsulation studies, giving insights into hydrophobic effects and pH-driven guest uptake.^4,7^

**Fig. 1:**
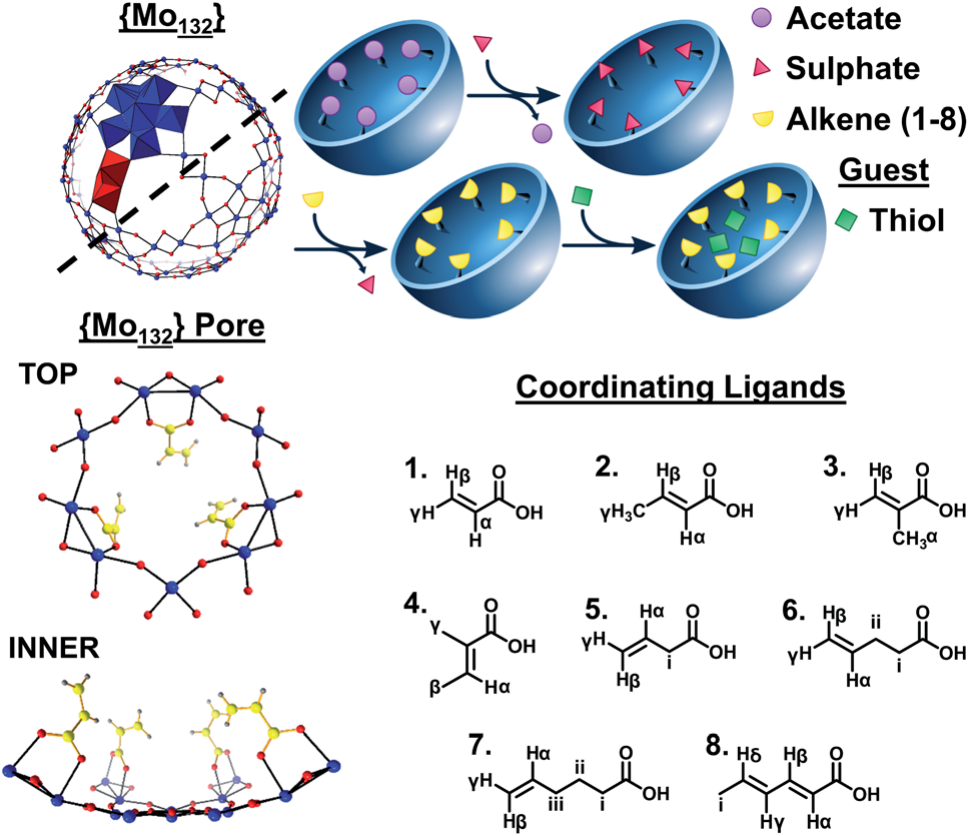
Simplified ball-and-stick representation of the {Mo_132_} framework highlighting the pentagonal (blue polyhedra) and linker (red polyhedra) structural units. An enhanced view of one of the 20 surface pores is shown, presenting the coordination mode of acrylate ligands at the inner surface. A simplified scheme of the overall ligand exchange process from {Mo_132_(OAc)_30_} to the formation of **1–8**, followed by the uptake of thiol guest species, is presented. Finally, the ligands used for the synthesis of **1–8** are shown, with labelled proton groups, as used for data analysis herein; L = acrylic acid, **1**; crotonic acid, **2**; methacrylic acid, **3**; tiglic acid, **4**; 3-butenoic acid, **5**; 4-pentenoic acid, **6**; 5-hexenoic acid, **7**; and sorbic acid, **8**. Atom key: Mo; blue, O; red, C; yellow, and H; grey.

Molybdenum-based Keplerate-type structures possess a metal-oxo framework containing 132 Mo atoms consisting of twelve {Mo_6_} pentagonal units and thirty {Mo_2_} linkers. The pentagonal and linker-type units (Fig. 1) are arranged to form 20 hexagonal {Mo_9_O_9_} flexible pore-like voids, with diameter *ca.* 3.6 Å, on the surface of the structure. The linker units are stabilized by the introduction of coordinating ligands, such as sulfates,^8^ oxalates,^9^ and carboxylate-containing species,^4,10^ which can replace coordinated water molecules on the inner surface of the structures.

Coordination of up to 30 ligands allows for the targeted design of the internal surface character. For example, the introduction of butyrate ions results in a hydrophobic internal surface, in contrast to the hydrophilic character expressed by the sulphate-containing structure.^11^ Compounds with the inorganic super-fullerene structure can be produced by either direct syntheses, from molybdate salts, or *via* the exchange of ligands present on a precursor Keplerate cluster with new donors. The ligand exchange process is facilitated by the porous nature of the framework with the apertures lending these structures sieve-like properties, preventing coordination of bulky ligands on the inner surface of the spheres.^12^

In contrast to the wide range of hydrophilic-ate ligands utilized in Keplerate synthesis, only a handful of, exclusively aliphatic, hydrophobic carboxylate-containing ligands have been incorporated into {Mo_132_}. We sought to coordinate hydrophobic π-systems, provided by alkene ligands, resulting in an electron-rich inner surface. Ligands were selected containing coordinating carboxylic acid groups along with alkene functionality with varied olefin regioisomers and alkyl tail length. Using direct and ligand exchange approaches we have thus synthesized, alkene-coordinated structures, **1–8** {(NH_4_)_42_[Mo_132_O_372_(L)_30_(H_2_O)_72_]} (L = (**1**) acrylic acid, (**2**) crotonic acid, (**3**) methacrylic acid, (**4**) tiglic acid, (**5**) 3-butenoic acid, (**6**) 4-pentenoic acid, (**7**) 5-hexenoic acid, and (**8**) sorbic acid), which offer new internal surface properties and are utilised to homogeneously encapsulate and stabilise short chain thiols, preventing their evaporation in an aqueous environment.

## Results and discussion

### Synthetic strategy

Two approaches for the synthesis of **1–8** were employed here: direct and ligand exchange syntheses. Firstly, **1** was obtained *via* a direct synthesis approach where the final product is synthesised from readily available starting materials. Adapting the long-established procedure for the direct synthesis of {Mo_132_(OAc)_30_}^6^ by replacing the acetate-containing reagents (acetic acid and ammonium acetate) with ammonia solution and an excess of acrylic acid, the ammonium salt of **1** was obtained. **1** could also be obtained by ligand-exchange syntheses from a pre-assembled {Mo_132_} complex, such as {Mo_132_(OAc)_30_} or {Mo_132_(SO_4_)_30_}. Use of the {Mo_132_(OAc)_30_} or {Mo_132_(SO_4_)_30_} precursors resulted in faster, higher-yielding product formation (4 days) than in the direct synthesis (7 days), while the {Mo_132_(SO_4_)_30_} ligand exchange method produced crystals of the greatest quality for XRD study. Ligand exchange from {Mo_132_(SO_4_)_30_} produced a further seven alkenyl-{Mo_132_} products, **2–8**, which were not isolable *via* direct methods.

### Structural analysis

**Fig. 2:**
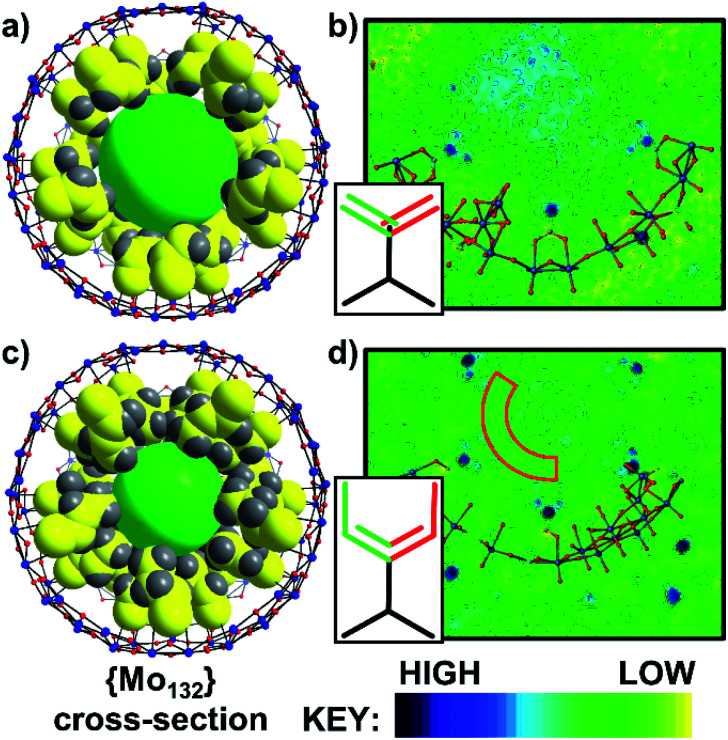
(a) Combined ball-and-stick and space-filling model of **1**, (b) 2D electron density map of **1**, (c) combined ball-and-stick and space-filling model of **2**, and (d) 2D electron density map of **2**. For (a) and (c) 1 pentagonal and 5 associated {Mo_2_} linker-type units have been removed for simplicity. The green sphere highlights the inner cavities of **1** and **2**. The electron density maps show the approximately even distribution between the two positions of the ligand tails, as highlighted by the green/red bonds shown in the insets. For **2**, there is a clear distinction between the ligand tail positions and the remaining unassigned electron density of the internal cavity. The region of low electron density between the ligand tails and internal cavity in **2** is partially highlighted by the dark red box in (d) and represents a hydrophobic region within the cavity with reduced water molecule occupancy. Atom key: Mo; blue, O; red, C; yellow, and H; grey.

The directly synthesised single crystal X-ray structure of **1** (Fig. 2a), exhibits an *R*3̄ space group and confirms the expected presence of the spherical {Mo_132_} framework. Additionally, 30 acrylate ligands are coordinated at the {Mo_2_} linkers, with their tails facing towards the core of the Keplerate. The electron density map of **1** (Fig. 2b) highlights that the distribution of electron density of the ligand tails between two positions, due to rotation, is approximately equal. The ligand tails create an internal cavity which is approximately 12 Å in diameter, with an overall volume of approximately 905 Å^3^. The internal cavities are not unoccupied, as indicated by the observed residual electron density peaks; however, this cannot be definitively refined as specific counterions or solvent molecules.

Full structural analysis of **2** and **5** confirmed that the {Mo_132_} framework is retained upon the ligand exchange synthesis. Coordinated alkene ligands, crotonic acid (**2**) and 3-butenoic acid (**5**) are found at the {Mo_2_} linker-type positions. The ligand tails hang towards the center of the structures, resulting in hydrophobic inner cavities of approximately 10 Å diameter, with a volume of approximately 523 Å^3^ (Fig. 2c). The cavity volumes for both are significantly lower than that for **1** due to the longer chain length of the ligands used here. For **2** and **5**, there is a distinct area of low electron density between the positions of the ligand tails and the electron density of the central cavity, caused by electrostatic interactions between the terminal protons of the ligand tails and the solvent water molecules (Fig. 2d and S28†). The shorter length of the ligands in **1** in comparison to those coordinated in **2** and **5** (approximately 4.4 Å *vs.* 5.5 Å) and the subsequently lower clustering of ligand tails towards the center of the cavity reduces this region of low electron density in the acrylate-coordinated structure.

For the remaining structures, the {Mo_132_} framework was initially confirmed using X-ray diffraction by comparison of the unit cell dimensions of the single crystals. The UV-Vis spectra for all structures contained a broad peak with *λ*_max_ at approximately 450 nm, due to the presence of reduced Mo^V^ centers, as expected for {Mo_132_} structures (Fig. S3†). For **2–8**, IR spectroscopy was utilized to confirm the complete replacement of sulphate ligands by the alkene ligands had occurred, with the absence of the typical triplet pattern, due to the splitting of the ν_3_ stretching mode of coordinated sulphate ligands, between 1040–1200 cm^−1^ (Fig. S2†). The observations established in XRD and IR studies are further facilitated by elemental analyses (Table S2†) and NMR spectroscopy solution studies, as will be discussed herein.

### NMR solution studies

Solutions of **1–8** in D_2_O (5 mM) were prepared for NMR study. For **1–8**, the information obtained from these measurements follows a similar general trend therefore, to simplify the presentation of results, only the NMR data for **5** will be discussed in detail here, as it produced clear and well-defined peaks in the resulting spectra. Extensive data and analyses for the remaining structures is presented in the ESI.†^13^

Measurements were carried out by dissolving crystalline samples of **1–8** in D_2_O, however, alternative approaches can be applied with amorphous material which gives broadly similar results. For example, using {Mo_132_(SO_4_)_30_}, which is ^1^H and ^13^C silent, we can directly detect the result of ligand coordination by addition of the appropriate alkene to the solution. The primary difference in using this approach is that the number of coordinated ligands is lower here than using pure product crystals, due to the lower number of ligands added overall. The {Mo_132_(OAc)_30_} structure can also be used in this manner; however, the presence of NMR active nuclei can obscure analyses.

**Fig. 3:**
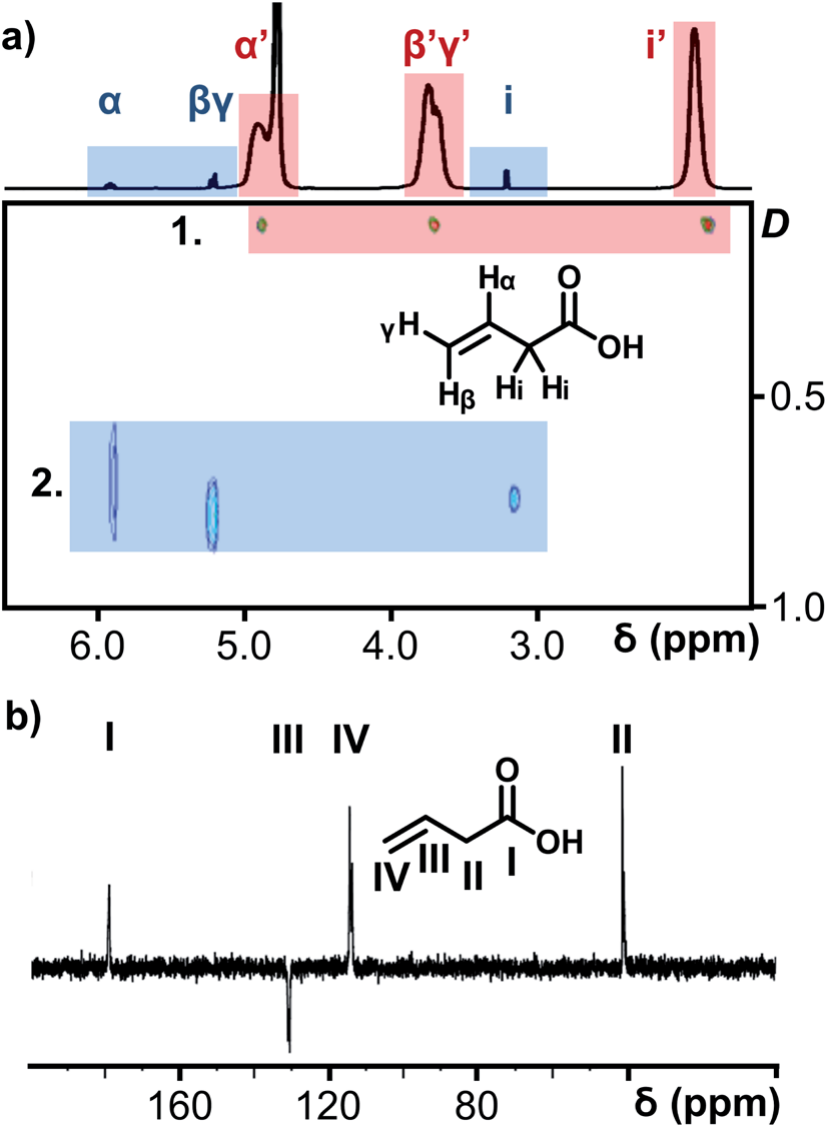
(a) ^1^H NMR spectrum of **5**, overlaid on the ^1^H DOSY NMR spectrum of **5** (units for diffusion coefficient, *D*: ×10^−9^ m^2^ s^−1^). The red box (marked 1.) highlights the diffusion coefficient of the encapsulated 3-butenoic acid ligands whilst the blue box (marked 2.) highlights those signals arising from the solvated free ligands. (b) ^13^C DEPTQ NMR spectrum of **5** for unambiguity in the assignment of carbon and proton nuclei for all ligands, a different labelling convention has been used for the carbon nuclei.

The porous nature of the {Mo_132_} structure facilitates partial ligand exchange with the solvent system (D_2_O), providing the ligands are sufficiently labile, upon crystal dissolution. The resulting solution therefore is expected to contain signals representing coordinated ligands and resonances for free ligand species. Critically, encapsulation in the negatively charged and electron dense {Mo_132_} structure leads to a separation of these peaks for a single ligand species which exists in two distinct domains.^14,15^ In this regard, the ^1^H NMR of **5** (Fig. 3), contains three small sharp resonances for the free 3-butenoic acid ligand: CH_2_(i) *δ* 3.2 ppm, CH_2_(β,γ) *δ* 5.3 ppm, and CH(α) *δ* 6.0 ppm, and 3 corresponding broad peaks for the coordinated 3-butenoic acid ligands: CH_3_(i′) *δ* 1.9 ppm, CH_2_(β′,γ′) *δ* 3.7 ppm, and CH(α′) *δ* 4.9 ppm, representing upfield peak shifts of Δ*δ* −1.9 ppm, −1.6 ppm and −1.1 ppm, respectively. The simultaneous presence of two sets of distinct peaks indicates that the exchange process is slow on the NMR timescale, with fast exchange regimes expected to result in a single peak set. Additional peaks at *δ* 4.8 ppm and *δ* 7.4 ppm arise from solvent water and NH_4_^+^ cation species, respectively.

Further NMR studies were utilised to validate the above observations. ^13^C NMR measurements of **5** (Fig. S31e†) followed a similar trend to the ^1^H NMR measurements. The carbon nuclei have been assigned sequentially, I–IV for **5**, from the carboxylate carbon, to differentiate the discussion of these nuclei from the proton assignments. Sharp peaks assigned to the carbon nuclei of the free, solvated ligands are observed at *δ* 179 ppm [–COO^−^(I)], *δ* 130 ppm [–CH(III)], *δ* 118 ppm [–CH_2_(IV)], and *δ* 41 ppm [–CH_2_(II)]. Broad peaks are also observed at the same positions as the sharp peaks, attributed to the coordination of the ligands within the {Mo_132_} structure, see Fig. S31e† for expanded spectra. Diffusion-Ordered Spectroscopy (DOSY) NMR has been employed to establish diffusion coefficients of the ligand species, which are inversely related to molecular size. The spectrum for **5** confirmed the presence of two distinct signals originating from free and encapsulated species, as shown in Fig. 3a. The broad peaks possess diffusion coefficients in the range of 110–115 pm^2^ s^−1^, whereas the free solvated ligand peaks, have higher diffusion coefficients in the range of 700–740 pm^2^ s^−1^ due to the former's coordination to the very large {Mo_132_} framework. The diffusion coefficients of the broad peaks relate to a hydrodynamic radius of ∼22 Å, which is consistent with the inner cavity of {Mo_132_} which has a crystallographic outer diameter of (∼32 Å) and with previously reported values,^16^ indicating that these peaks originate from internally bound ligands, while a large difference in the two diffusion coefficients obtained indicate two distinct domains for the ligands – coordinated inside the framework and free, solvated small molecules outside the Keplerate sphere.

### Thiol uptake studies

The encapsulation of guest species within a suitable host provides a potential environment to protect against mechanical stress, temperature changes, reduction and oxidation. The use of porous materials to encapsulate guest species has been applied to various host classes such as extended arrays (MOFs,^17^ zeolites^18^), mesoporous materials,^19^ porous liquids,^20^ and organic materials.^21–23^ Whilst organic systems are commonly studied using solution state methods, for inorganic hosts, heterogenous mixtures are more widely used to form the host–guest complex, with product analyses typically performed on the resulting solid material. Previous studies have utilised {Mo_132_} frameworks, with alternative ligands, as hosts, for example to uptake alcohols using {Mo_132_} decorated with propionate ligands.^4^ Here, we sought to use the {Mo_132_} alkene-containing structures to promote the homogeneous uptake of volatile organic species from aqueous solution, resulting in a stable host–guest complex which protects against guest evaporation at elevated temperatures.

Short-chain alkyl thiols (R–SH), are volatile species which are structurally analogous to alcohols, with –SH replacing the hydroxyl group. The large size of the sulfur atom makes it more polarizable than oxygen, while the reduced electronegativity of sulphur results in weaker hydrogen and intermolecular bonding, compared to the analogous alcohols, as reflected in the lower boiling points of the thiol species. Alkyl thiols are also of interest for their potential to undergo thiol-ene type reactions with the alkene functional group of the coordinated ligand species.^24^ To explore the uptake of alkyl thiols, we utilized alkenyl appended Keplerates **1–8** to facilitate the uptake of a series of alkyl thiols from aqueous solutions. For all measurements, solutions of {Mo_132_(SO_4_)_30_} in D_2_O (5 mM) were prepared in NMR tubes, followed by addition of excess ligand and guest. Observations will be described in detail for NMR interpretation using the 3-butenoic acid ligand, as described for **5** above, although comparison studies indicate the trends observed are applicable to all ligands used in **1–8**.

Two sets of alkyl thiol isomers, with maximum chain lengths of 3–4 carbons were selected as guests to monitor their interaction with the {Mo_132_} host. Propanethiol and butanethiol differ only by their carbon chains and their branched isomers were selected to monitor any structural effects on uptake capacities. Explicitly, the isomers used in this study are 1-propanethiol (**A**), 2-propanethiol (**B**), 1-butanethiol (**C**), 2-butanethiol (**D**), 2-methyl-1-propanethiol (**E**), and 2-methyl-2-propanethiol (**F**). For brevity we will focus on guest **C** as the benchmark for these studies.

**Fig. 4:**
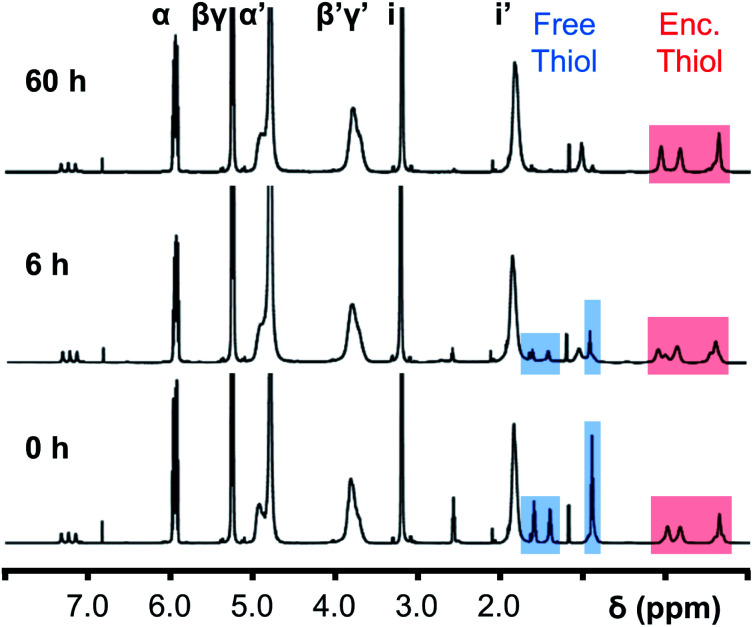
Comparison of {Mo_132_(SO_4_)_30_} with added 3-butenoic acid ligands and 1-butanethiol guests (**C**) prior to and post heating to 70 °C for 6 and 60 h. The peaks associated with the solvated 1-butanethiol guest (blue box) significantly reduce after 6 h until no signal is observed after 60 h. In contrast, the resonances arising from encapsulated 1-butanethiol (red box) remain largely unchanged after the same period.

Initially, 60 equivalents of the thiol guests were added to a solution of {Mo_132_(SO_4_)_30_} containing alkene ligands (also 60 equivalents) in D_2_O, at room temperature. Addition of the thiol species under these conditions resulted in their immediate uptake, as indicated by the presence of broadened peaks appearing between *δ* −0.6 ppm and 0.0 ppm, displacing internal solvent molecules, in addition to solvated thiol peaks (*δ* 0.9–1.6 ppm) (Fig. 4). To confirm that the origin of the broad peaks is from the encapsulation of the thiol species, additional NMR experiments were performed which could be interpreted in a similar manner to those which were used for the previously described structural characterizations, namely ^13^C, DEPTQ, HSQC, and DOSY spectra (Fig. S56, S57 and S59†).

Analysis of the extent of encapsulation could be determined by use of a methanesulfonic acid external reference (Table S6†). The data obtained indicates that the extent of encapsulation is dominated by steric crowding of the central cavity of {Mo_132_}, with longer ligand alkyl tails resulting in decreased uptake of the thiol guests. Additionally, the bulkier 2-methyl-2-propanethiol species (**F**) is broadly prevented from entering the inner cavity due to its large size in comparison to the {Mo_132_} pore, which restricts internal access. To confirm that the addition of the thiol guests does not result in thiol oxidation and replacement of the previously coordinated alkene ligands, a comparison of the peak intensities for the alkene ligands prior to and following the addition of thiol guest was carried out. Fig. S58† shows that there is no significant decrease in the integral of the encapsulated alkene ligands or increase in the peak height for the solvated alkene ligands, as would be anticipated upon their replacement, indicating that the thiol species are contained within the central cavity of the {Mo_132_} structure.

Previous analogous encapsulation studies have utilised elevated temperatures to promote increased guest encapsulation and a similar investigation was applied here.^25^ Upon heating samples to 70 °C, during NMR analysis, an increase in the ratio of the encapsulated thiol species was coupled with a corresponding increase in the ratio of the free thiol species, with these initial changes attributed to the improved solubilities of the thiol species at elevated temperature. Continuous heating of the sample at 70 °C leads to a significant decrease in the peak intensity of the signals attributed to the free thiol species after a period of 6 h with almost no free thiol remaining after a period of heating of 60 h (Fig. 4). This behavior is reflected in the ^1^H and ^13^C NMR spectra of guest **C** in D_2_O (Fig. S60a–f and S61†), with no additional peaks, representing side-products, being observed in any spectra. Similar measurements carried out using a J-Young's tap NMR tube led to no significant change in the observed peak heights even after 40 h at elevated temperature, with subsequent heating of the sample for 20 h without a cap resulting in a loss of most of the NMR signal (Fig. S63†). These results indicate that heating of the 1-butanethiol in D_2_O results in the loss of the species by evaporation.

The changes observed for the free solvated thiol species are not reflected in the peak intensities of the signals attributed to the encapsulated species, even after elevating the temperature to 95 °C for 60 min (Fig. S64†). This is confirmed using HSQC before and after heating, with only the loss of signals related to the free thiol species observed (Fig. S62†). This indicates that the clustering of the thiol species within the {Mo_132_} cavity structure results in effective thermal insulation, preventing evaporation of even the most volatile encapsulated thiol species (**E**, b.p. = 70 °C) from occurring. Additionally, the initially established equilibrium, with free thiol and encapsulated thiol co-existing, is not re-established after heating due to the preferential clustering of the hydrophobic thiol species within the hydrophobic cavity.

Comparison of uptake in the system described above with only {Mo_132_(SO_4_)_30_}, and {Mo_132_(SO_4_)_30_} + 60 acetate ligands, was performed. The resulting NMR spectra showed no uptake of any of the thiol species (Fig. S52c and d† for guest **C**), with peaks present for the free thiol guests only, except for minimal uptake of **B** and **D** with added acetate ligands. Upon increasing the number of equivalents of acetate added, from 60 to 90 and 120 equivalents, broadened peaks were observed, as described with alkene ligands, with reduced peak intensity here. By measuring the number of coordinated alkene or acetate ligands in each scenario, an understanding of this behavior can be derived. With 60 equivalents of ligand added, for 3-butenoic acid, the number of coordinated ligands is approximately 26 (with 34 solvated free ligands), while, for acetate, the number of coordinated ligands is approximately 13 (with 50 solvated free ligands). Increasing the number of equivalents of acetate added results in 15 coordinated ligands with 90 equivalents and 16 coordinated ligands with 120 equivalents added (Table 1). Therefore, the inner surface of {Mo_132_} with added alkene ligands is decorated with a much higher number of ligands than for the same conditions with acetate, resulting in a cavity surface with increased hydrophobicity, due to the hydrophobic alkyl/alkene tails. This hydrophobic character enables the encapsulation of the primarily hydrophobic thiol guests at comparably lower concentrations of alkene in comparison to the acetate ligands. This encapsulation behaviour is not observed at all with only the hydrophilic {Mo_132_(SO_4_)_30_} structure, with no added hydrophobic components (acetate ligands or the ligands used for **1–8**), (Fig. S52c†) due to the hydrophilic nature of the internal cavity promoted by the coordinated sulphate ligands, further confirming the role of the hydrophobic interaction in facilitating uptake.

**Table 1:** Comparison of the extent of ligand coordination with 60, 90, and 120 equivalents of either acetate or 3-butenoic acid ligands added. The resonances attributed to encapsulated 1-butanethiol (**C**) are highlighted by the red boxes

Added ligand	Acetate		3-Butenoic acid	
	Coord. ligand	Enc. guest^a^	Coord. ligand	Enc. guest^a^
60	13.3	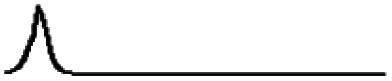	25.5	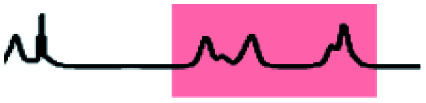
90	15.4	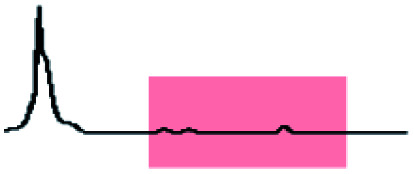	25.8	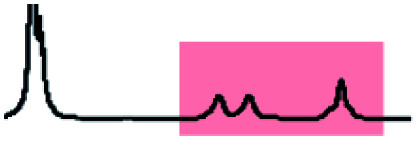
120	16.4	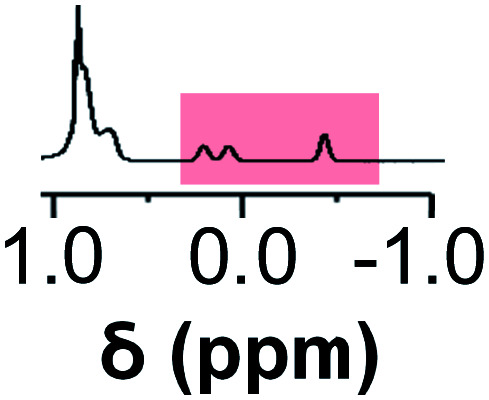	26.1	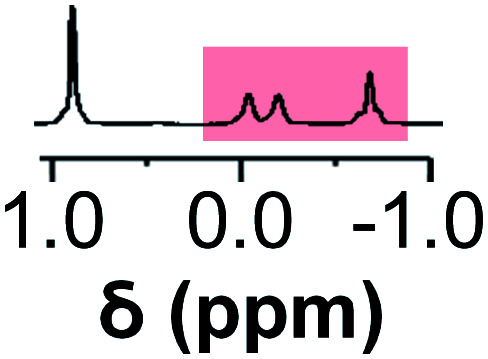

a 1-Butanethiol.

## Conclusions and future work

Keplerate POMs, based on the {Mo_132_} framework, have been appended with alkenyl carboxylate ligands, through ligand exchange from {Mo_132_(SO_4_)_30_} creating an electron rich hydrophobic cavity measuring up to 12 Å in diameter. X-ray and NMR studies have demonstrated that alkenyl carboxylate uptake is higher than for acetate ligands creating a more hydrophobic cavity than previously observed. Short chain alkyl thiol uptake within the Keplerate cavity in aqueous media was demonstrated by NMR studies and shown to provide additional thermal insulation to the guest thiols, potentially allowing for novel higher temperature reactivity in future work.

## Conflicts of interest

The authors declare no competing financial interests.

## Supplementary Material

SC-011-C9SC06217C-s001

SC-011-C9SC06217C-s002
