# Supplementary material for: Embedding alkenes within an icosahedral inorganic fullerene {(NH4)42[Mo132O372(L)30(H2O)72]} for trapping volatile organics
Source: Chem Sci. 2020 Jan 23;11(9):2388–93. doi: 10.1039/c9sc06217c (PMC8157328; doi:10.1039/c9sc06217c)
Supplement: SC-011-C9SC06217C-s001 [file SC-011-C9SC06217C-s001.pdf]

Electronic Supplementary Material (ESI) for Chemical Science.
This journal is © The Royal Society of Chemistry 2020

# SUPPORTING INFORMATION

## Embedding Alkenes within an Icosahedral Inorganic Fullerene $\{(NH_4)_{42}[Mo_{132}O_{372}(L)_{30}(H_2O)_{72}]\}$ for Trapping Volatile Organics

Robert W. Pow, Weimin Xuan, De-Liang Long, Nicola L. Bell, and Leroy Cronin*

School of Chemistry, University of Glasgow, University Avenue, Glasgow, G12 8QQ, United Kingdom

**Contents**

(1) Previously Reported Structures ..... 3

(2) Materials and Methods ..... 4

CHN Microanalysis ..... 4

Nuclear Magnetic Resonance Spectroscopy ..... 4

Single Crystal X-Ray Diffraction ..... 4

pH Measurements ..... 4

Infrared Spectroscopy ..... 4

Thermogravimetric Analysis ..... 4

(3) Synthetic Procedures ..... 4

Synthesis of $\{Mo_{132}(OAc)_{30}\}$ for Ligand-Exchange Syntheses and $^1$H NMR Ligand-Exchange Studies ..... 4

Synthesis of $\{Mo_{132}(SO_4)_{30}\}$ from $\{Mo_{132}(OAc)_{30}\}$ for use in Ligand-Exchange Syntheses ..... 5

Direct Synthesis of $\{Mo_{132}$(Acrylic acid)$\}$ ..... 5

Ligand-Exchange Syntheses of 1-8 from $\{Mo_{132}(SO_4)_{30}\}$ ..... 5

(4) Structural Formulae ..... 6

(5) Thiol Uptake Procedure ..... 7

(6) Infrared Spectroscopy of 1-8 ..... 7

(7) UV-Vis Spectroscopy of 1-8 ..... 8

(8) Elemental Analysis of 1-8 ..... 9

(9) Crystallographic Data of 1, 2, and 5 ..... 9

(10) Characterization of 1 – $\{Mo_{132}$(Acrylic acid)$\}$ ..... 12

(10.1) Thermogravimetric Analysis of 1 ..... 13

(10.2) NMR Study of 1 .......... 13
(11) Characterization of 2 – {$Mo_{132}$(Crotonic acid)} .......... 17
(11.1) Thermogravimetric analysis of 2 .......... 17
(11.2) NMR Study of 2 .......... 18
(12) Characterization of 3 – {$Mo_{132}$(Methacrylic acid)} .......... 20
(12.1) Thermogravimetric Analysis of 3 .......... 20
(12.2) NMR Study of 3 .......... 20
(13) Characterization of 4 – {$Mo_{132}$(Tiglic acid)} .......... 22
(13.1) Thermogravimetric Analysis of 4 .......... 22
(13.2) NMR Study of 4 .......... 23
(14) Characterization of 5 – {$Mo_{132}$(3-Butenoic acid)} .......... 25
(14.1) Thermogravimetric Analysis of 5 .......... 26
(14.2) NMR Study of 5 .......... 26
(15) Characterization of 6 – {$Mo_{132}$(4-Pentenoic acid)} .......... 29
(15.1) Thermogravimetric Analysis of 6 .......... 29
(15.2) NMR Study of 6 .......... 29
(16) Characterization of 7 – {$Mo_{132}$(5-Hexenoic Acid)} .......... 31
(16.1) Thermogravimetric Analysis of 7 .......... 31
(16.2) NMR Study of 7 .......... 32
(17) Characterization of 8 – {$Mo_{132}$(Sorbic Acid)} .......... 34
(17.1) Thermogravimetric Analysis of 8 .......... 34
(17.2) NMR Study of 8 .......... 34
(18) Thiol-containing Guest Uptake .......... 36
Investigation of Uptake between Pure {$Mo_{132}(SO_4)_{30}$}, {$Mo_{132}(SO_4)_{30}$} plus acetic acid, and {$Mo_{132}(SO_4)_{30}$} plus alkene ligands, with Thiol-Containing Alkyl Guests .......... 36
Investigation of Increasing Temperature on Uptake of 1-butanethiol with {$Mo_{132}(SO_4)_{30}$} plus 3-butenoic acid . 46
References .......... 50

## (1) Previously Reported Structures

**Table S1** Reported {$Mo_{132}$} ligands and their associated counterions.

| Ligand | Structure | Counterions | | |
|---|---|---|---|---|
| Acetate | 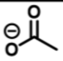 | $NH_4^+$ [1] | $NH_4^+$/DODA$^+$ [a] [2] | $NH_4^+$/MimC$_n^+$ [b] [3] |
| | | $Na^+$/DMA$^+$ [c] [4] | $Na^+$/MimAm$^+$ [d] [5] | |
| Sulfate | 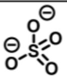 | $NH_4^+$ [3] | $NH_4^+$/$Cs^+$ [6] | $NH_4^+$/$Ce^+$ [6] |
| | | $NH_4^+$/$Pr^{3+}$ [7] | $NH_4^+$/$Li^+$ [8] | $NH_4^+$/DODA$^+$ |
| | | $NH_4^+$/Formadinium [9] | $NH_4^+$/$Ca^{2+}$/ Formadinium [9] | $NH_4^+$/Formadinium/ $Pr^{3+}$ [10] |
| | | $NH_4^+$/Formadinium/ $Gd^{3+}$ [11] | $NH_4^+$/Urea-$H^+$ [12] | $NH_4^+$/Urea-$H^+$/$Ca^{2+}$ [12] |
| | | $Na^+$/Urea-$H^+$ [6] | $Li^+$/DMA$^+$ [8] | DMA$^+$/$Al^{3+}$ [13] |
| Phosphate | 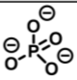 | $NH_4^+$ [6] | $NH_4^+$/$Na^+$ [6] | |
| Acetate/Sulfate mix | | $NH_4^+$ [14] | $Na^+$/DMA$^+$ [14] | |
| Sulfate/Phosphate mix | | Guanadinium [6] | | |
| Formate | 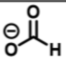 | $NH_4^+$ [15] | Formadinium [15] | |
| Carbonate | 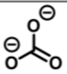 | $NH_4^+$ [16] | $NH_4^+$/Formadinium [16] | |
| Oxalate | 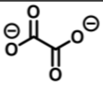 | $NH_4^+$ [17] | | |
| Propanoate | 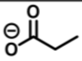 | $NH_4^+$ [18] | | |
| Butyrate | 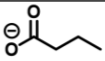 | $NH_4^+$ [19] | | |
| *iso*-Propanoate | 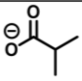 | $Na^+$ [20] | | |
| *tert*-Butyrate | 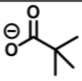 | $Na^+$ [20] | | |
| L-Tartrate | 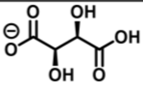 | $NH_4^+$ [21] | $NH_4^+$/DMA$^+$ [21] | TBA$^+$ [e] [21] |
| D-Tartrate | 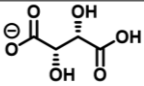 | $NH_4^+$ [21] | DMA$^+$ [21] | |
| Succinate | 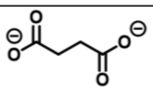 | $NH_4^+$ [22] | $NH_4^+$/DMA$^+$ [22] | |
| Glutarate | 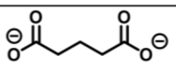 | $NH_4^+$ [22] | $NH_4^+$/DMA$^+$ [22] | |

[a] Dioctadecyl-dimethylammonium. [b] 1-methyl-3-alkylimidazolium (where, $n$ = 12, 14, 16, 18, 20). [c] Dimethyl ammonium.
[d] 3-aminoethyl-1-methylimidazolium. [e] $n$ (tetrabutylammonium).

## (2) Materials and Methods

All chemicals and solvents were used as purchased without further purification.

***CHN Microanalysis*:** Carbon, nitrogen and hydrogen content were determined by the microanalysis services within the School of Chemistry, University of Glasgow using an EA 1110 CHNS, CE-440 Elemental Analyzer.

***Nuclear Magnetic Resonance Spectroscopy*:** $^{1}H$ NMR spectra were recorded on a Bruker Ascend Aeon 600 MHz NMR spectrometer. Samples were analysed in Bruker NMR tubes (5 mm diameter, 7" length). Unless otherwise stated, $1.7 \times 10^{-3}$ M solutions of the $\{Mo_{132}\}$-based structures were prepared by dissolving the relevant sample in $D_2O$. Where required, a methanesulfonic acid reference solution of concentration $3.4 \times 10^{-2}$ M (i.e. 20x the concentration of the $\{Mo_{132}\}$ solution) was prepared and transferred to an external axial reference tube.

***Single Crystal X-Ray Diffraction*:** A suitable single crystal was selected and mounted onto a rubber loop using Fomblin oil. Single-crystal datasets and unit cells were collected at 150(2) K on a Bruker Apex II Quasar diffractometer equipped with a graphite monochromator ($\lambda$ ($Mo_{K\alpha}$) = 0.71073 Å) equipped with a microfocus X-ray source (50 kV, 1.0 mA) or at Diamond Light Source on Beamline I19 under proposal MT18953. Data collection and reduction were performed using the Apex2 software package and structure solution and refinement were carried out with SHELXS-97[23] and SHELXL-97[24] or later version via WinGX[25]. All of the non-hydrogen atoms (including those disordered) were anisotropically refined. CCDC 1962975-1962977 contains the crystallographic data for structures **1**, **2**, and **5**, and can be obtained free of charge via www.ccdc.cam.ac.uk/data_request/cif, or by emailing data_request@ccdc.cam.ac.uk, or by contacting The Cambridge Crystallographic Data Centre, 12 Union Road, Cambridge, CB2 1EZ, UK; fax: +44 1223 336033.

***pH Measurements*:** Measurements were taken on a Hanna Instruments HI-2210-02 Bench Top pH Meter with pH electrode (HI 1131B) and temperature probe (HI 7662).

***Infrared Spectroscopy*:** All samples were collected in transmission mode using a JASCO FT-IR-410 spectrometer.

***Thermogravimetric Analysis*:** Analysis was performed on a TA Instruments Q 500 Thermogravimetric analyser under nitrogen air flow with a heating rate of 10 °C $min^{-1}$ from room temperature (22 °C) to 1000 °C.

## (3) Synthetic Procedures

### *Synthesis of $\{Mo_{132}(OAc)_{30}\}$ for Ligand-Exchange Syntheses and $^{1}H$ NMR Ligand-Exchange Studies*[1]

Ammonium heptamolybdate tetrahydrate $[(NH_4)_6Mo_7O_{24}.4H_2O]$ (5.6 g, 4.5 mmol) and ammonium acetate $[CH_3COONH_4]$ (12.5 g, 162 mmol) were added to a wide-necked 500 mL conical flask containing deionised water (250 mL) and a magnetic stirrer bar. Acetic acid $[CH_3COOH]$ (50%) was added, under stirring, until the solution

reached pH 4 (83 mL). Finally, hydrazine sulphate [$N_2H_4.H_2SO_4$] (0.8 g, 6.1 mmol) was added and the solution was stirred at room temperature for 10 minutes. The resulting dark brown solution was transferred to a crystallisation space (18 °C) and left uncovered to allow solvent evaporation to occur. After 2 days, dark brown hexagonal crystals had formed which were suitable for single-crystal X-ray diffraction data collection – used here to confirm the expected unit cell of the {$Mo_{132}$} structure. The crystals were collected and washed with a minimum amount of ethanol followed by diethyl ether, then dried in air. Yield: 4.04 g (60.6 %, based on Mo).

***Synthesis of {$Mo_{132}(SO_4)_{30}$} from {$Mo_{132}(OAc)_{30}$} for use in Ligand-Exchange Syntheses***[26]

{$Mo_{132}(OAc)_{30}$} (4.0 g, 0.14 mmol), prepared as above, and ammonium sulphate [$(NH_4)_2SO_4$] (16.0 g, 121.1 mmol) were added to a wide-necked 500 mL conical flask containing deionised water (320 mL) and a magnetic stirrer bar. Addition of hydrochloric acid [HCl] (16%), under stirring, produced a solution with pH 1 (16 mL), which was then stirred at 90 °C for 4 hours. After stirring, the dark brown solution was filtered whilst hot, and the filtrate was transferred to the crystallisation room (18 °C) for storage and left uncovered to allow for solvent evaporation to occur. After 4 days, dark brown hexagonal crystals had formed which were suitable for single-crystal X-ray diffraction data collection – used here to confirm the expected unit cell of the {$Mo_{132}$} structure. The crystals were collected and washed with a minimum amount of ethanol followed by diethyl ether, then dried in air. Yield: 1.03 g (25.8 %).

***Direct Synthesis of {$Mo_{132}$(Acrylic acid)}***

Ammonium heptamolybdate tetrahydrate [$(NH_4)_6Mo_7O_{24}.4H_2O$] (1.08 g, 1.0 mmol) was added to a 100 mL conical flask containing deionised water (75 mL) and a magnetic stirrer bar. Addition of ammonia solution (25%) (2 mL), acrylic acid (7 mL, 102.0 mmol), and sodium dithionite [$Na_2S_2O_4$] (85%) (140 mg, 0.8 mmol) followed. The solution was stirred at room temperature for 12 hours, followed by filtration to remove the small amount of precipitate which had formed in the dark brown solution. The resulting solution was covered with parafilm and kept in storage (18 °C). After 2 days, the formation of a small amount of precipitate was removed again by filtration, and the solutions were sealed using parafilm for storage (18 °C). After a further 7 days, dark brown hexagonal crystals formed which were suitable for X-ray diffraction studies. The crystals were collected and washed with a minimum amount of ethanol followed by diethyl ether, then left to dry in air. Yield = 0.17 g (11.5 %, based on Mo).

***Ligand-Exchange Syntheses of 1-8 from {$Mo_{132}(SO_4)_{30}$}***

{$Mo_{132}(SO_4)_{30}$} (230 mg, $8.0 \times 10^{-3}$ mmol), prepared as above, was added to a wide-necked 50 mL conical flask containing deionised water (15 mL) and a magnetic stirrer bar. The desired ligand for use in each reaction (**Figure S1**) was added in the following amounts: crotonic acid (**2**) – 645 mg, 7.5 mmol (750 eq.), methacrylic acid (**3**) – 160 mg (150 μL), 1.8 mmol (180 eq.), tiglic acid (**4**) – 120 mg, 1.2 mmol (150 eq.), 3-butenoic acid (**5**) – 172 mg (169 μL), 2.0 mmol (250 eq.), 4-pentenoic acid (**6**) – 200 mg (204 μL), 2.0 mmol (250 eq.), 5-hexenoic acid (**7**) – 137 mg (140 μL), 1.2 mmol (150 eq.), and sorbic acid (**8**) – 134 mg, 1.2 mmol (150 eq.). The number of equivalents in brackets is the approximate number of equivalents of ligand added in comparison to the number of moles of {$Mo_{132}(SO_4)_{30}$}.

After stirring at 50 °C for 3 days, ammonium chloride ($NH_4Cl$) – 230 mg, 4.3 mmol, was added and the solutions were stirred for a further 10 mins. The solutions were then transferred to the crystallisation room (18 °C) for storage in their uncovered containers, to allow solvent evaporation to occur. Dark brown, hexagonal crystals which were suitable for single-crystal X-ray analysis appeared after a period of 4 days. The crystals were washed with a minimum amount of ethanol followed by diethyl ether, then dried in air. Yield = 70-90 mg (30-40%).

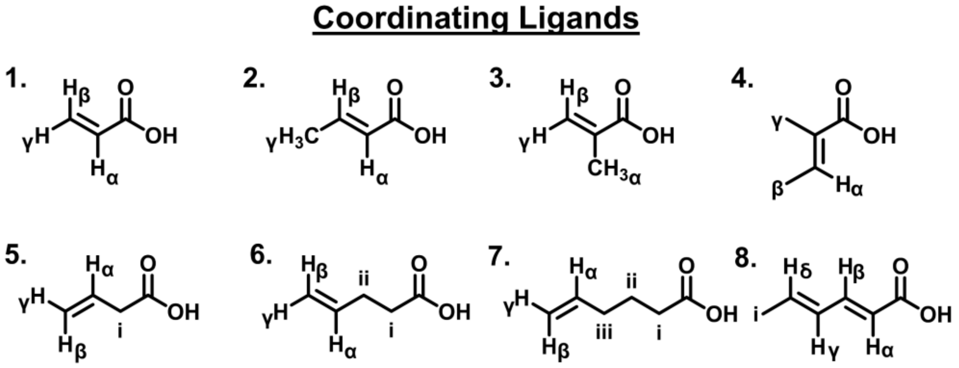


**Figure S1:** Ligands used for the synthesis of **1**-**8**: acrylic acid (**1**), crotonic acid (**2**), methacrylic acid (**3**), tiglic acid (**4**), 3-butenoic acid (**5**), 4-pentenoic acid (**6**), 5-hexenoic acid (**7**), and sorbic acid (**8**). Analysis of NMR spectra throughout the Supporting Information refers to the labelling of protons as indicated here. In the case of $^{13}C$ NMR studies the relevant carbon nuclei are labelled unambiguously.

**(4) Structural Formulae**

$(NH_4)_{42}[Mo_{132}O_{372}(C_3H_3O_2)_{22}(C_6H_7O_4)_8(H_2O)_{72}]$ · ca. 180 $H_2O$ + $[(NH_4)_2SO_4]_2$ = **1**

$(NH_4)_{42}[Mo_{132}O_{372}(C_4H_5O_2)_{30}(H_2O)_{72}]$ · ca. 160 $H_2O$ + $[(NH_4)_2SO_4]_3$ = **2**

$(NH_4)_{42}[Mo_{132}O_{372}(C_4H_5O_2)_{30}(H_2O)_{72}]$ · ca. 210 $H_2O$ + $[(NH_4)_2SO_4]_2$ = **3**

$(NH_4)_{42}[Mo_{132}O_{372}(C_5H_7O_2)_{30}(H_2O)_{72}]$ · ca. 80 $H_2O$ + $[(NH_4)_2SO_4]_3$ = **4**

$(NH_4)_{42}[Mo_{132}O_{372}(C_4H_5O_2)_{30}(H_2O)_{72}]$ · ca. 220 $H_2O$ + $[(NH_4)_2SO_4]_3$ = **5**

$(NH_4)_{42}[Mo_{132}O_{372}(C_5H_7O_2)_{30}(H_2O)_{72}]$ · ca. 90 $H_2O$ + $[(NH_4)_2(SO_4)]$ = **6**

$(NH_4)_{42}[Mo_{132}O_{372}(C_6H_9O_2)_{30}(H_2O)_{72}]$ · ca. 200 $H_2O$ + $[(NH_4)_2SO_4]$ = **7**

$(NH_4)_{42}[Mo_{132}O_{372}(C_6H_7O_2)_{30} (H_2O)_{72}]$ · ca. 190 $H_2O$ + $[(NH_4)_2SO_4]_3$ = **8**

### (5) Thiol Uptake Procedure

$\{Mo_{132}(SO_4)_{30}\}$ (50 mg, 1.7 x $10^{-3}$ mmol), prepared as above, was dissolved in $D_2O$ (0.6 mL) and added to an NMR tube (5 mm diameter, 7" length). Ligands and thiol guests were added, with shaking, with concentrations as a factor of a specific number of equivalents in comparison to the concentration of the $\{Mo_{132}(SO_4)\}$ (described explicitly in the relevant text). After 1 minute the samples were ready to be analyzed via NMR.

### (6) Infrared Spectroscopy of 1-8

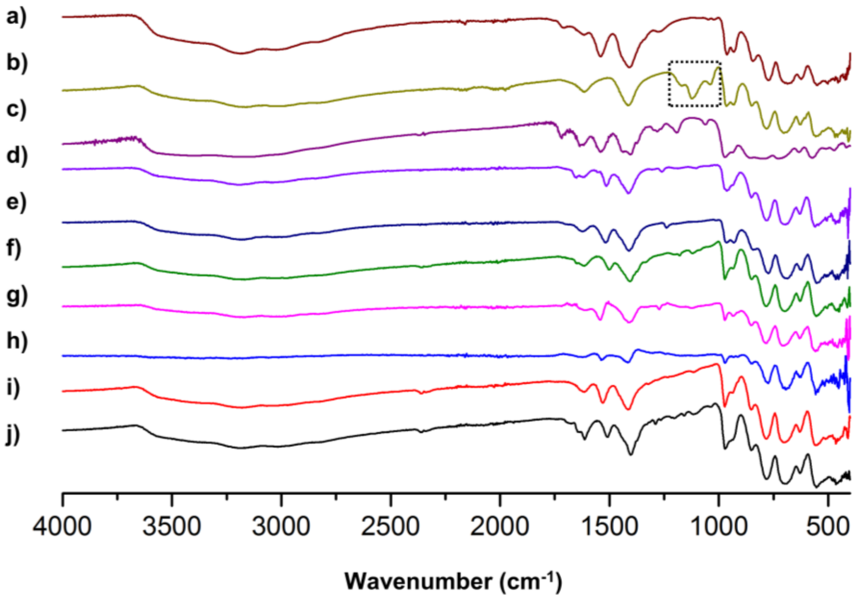


**Figure S2:** IR spectra for a) $\{Mo_{132}(OAc)_{30}\}$, b) $\{Mo_{132}(SO_4)_{30}\}$, c) **1**, d) **2**, e) **3**, f) **4**, g) **5**, h) **6**, i) **7**, and j) **8**. The triplet due to splitting of the $v_3$ stretching mode of the bidentate $SO_4^{2-}$ ligands ($C_{2v}$) in the 1030-1190 $cm^{-1}$ range, for $\{Mo_{132}(SO_4)_{30}\}$, is highlighted by the dashed black box.

**(7) UV-Vis Spectroscopy of 1-8**

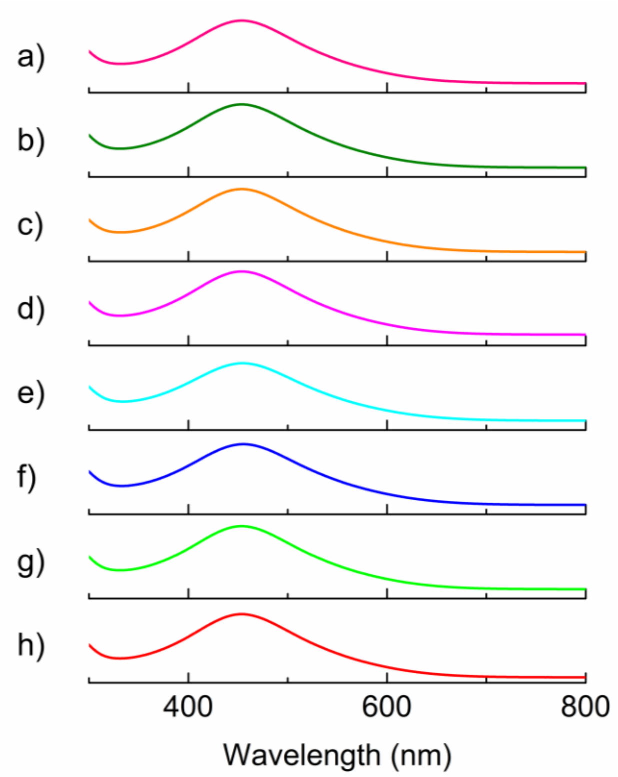


**Figure S3:** UV-Vis spectra for a) **1**, b) **2**, c) **3**, d) **4**, e) **5**, f) **6**, g) **7**, and h) **8**.

## (8) Elemental Analysis of 1-8

**Table S2** C, H, N, Mo, and S analysis for products **1-8**. All Values are given as percentages.

| STRUCTURE | C | H | N | Mo | S |
|---|---|---|---|---|---|
| 1 | 5.59 | 2.76 | 2.78 | 48.07 | 0.23 |
| 2 | 5.96 | 2.74 | 2.85 | 48.50 | 0.37 |
| 3 | 6.45 | 2.89 | 2.11 | 46.90 | 0.50 |
| 4 | 6.08 | 2.96 | 2.65 | 47.55 | 0.90 |
| 5 | 4.75 | 2.96 | 3.94 | 47.30 | 0.35 |
| 6 | 6.62 | 3.05 | 2.49 | 46.00 | 0.13 |
| 7 | 6.80 | 3.17 | 2.15 | 48.28 | 0.17 |
| 8 | 6.10 | 2.96 | 2.33 | 44.23 | 0.90 |

## (9) Crystallographic Data of 1, 2, and 5

**Table S3** Crystallographic data of **1** – {$Mo_{132}$(Acrylic Acid)}

| | | |
|---|---|---|
| Identification code | **1** | |
| Empirical formula | C114 H810 Mo132 N46 O708 S2 | |
| Formula weight | 26886.23 | |
| Temperature (K) | 150(2) | |
| Wavelength (Å) | 0.71073 | |
| Crystal system | Trigonal | |
| Space group | R-3 | |
| Unit cell dimensions | a = 32.6262(18) Å | α= 90° |
| | b = 32.6262(18) Å | β= 90° |
| | c = 73.225(5) Å | γ = 120° |
| Volume (Å$^3$), Z | 67503(9), 3 | |
| Density (calculated) (mg/m$^3$) | 1.984 | |
| Absorption coefficient (mm$^{-1}$) | 1.879 | |
| F(000) | 39168 | |
| Crystal size (mm$^3$) | 0.100 x 0.100 x 0.100 | |
| θ range for data collection (°) | 1.927 to 26.000 | |
| Limiting indices | -40<=h<=39, -40<=k<=40, -90<=l<=90 | |
| Reflections collected | 197269 | |
| Independent reflections | 29487 [R(int) = 0.0344] | |
| Completeness to theta | 25.242°/99.9% | |
| Absorption correction | Empirical | |
| Max. and min. transmission | 0.426 and 0.328 | |
| Refinement method | Full-matrix least-squares on F$^2$ | |
| Data / restraints / parameters | 29487 / 147 / 1400 | |
| Goodness-of-fit on F$^2$ | 1.152 | |
| Final R indices [I>2sigma(I)] | R1 = 0.0591, wR2 = 0.1527 | |
| R indices (all data) | R1 = 0.0775, wR2 = 0.1884 | |
| Extinction coefficient | n/a | |
| Largest diff. peak and hole (e. Å$^{-3}$) | 2.09 and -1.19 | |

**Table S4** Crystallographic data of **2** – {$Mo_{132}$(Crotonic acid)}

| | | |
|---|---|---|
| Identification code | **2** | |
| Empirical formula | C120 H806 Mo132 N48 O676 S3 | |
| Formula weight | 26502.34 | |
| Temperature (K) | 150(2) | |
| Wavelength (Å) | 0.6889 | |
| Crystal system | Trigonal | |
| Space group | R-3 | |
| Unit cell dimensions | a = 32.3898(3) Å | $\alpha$= 90° |
| | b = 32.3898(3) Å | $\beta$= 90° |
| | c = 72.8225(7) Å | $\gamma$ = 120° |
| Volume (Å$^3$), Z | 66162.6(14), 3 | |
| Density (calculated) (mg/m$^3$) | 1.995 | |
| Absorption coefficient (mm$^{-1}$) | 1.915 | |
| F(000) | 38586 | |
| Crystal size (mm$^3$) | 0.100 x 0.100 x 0.050 | |
| θ range for data collection (°) | 1.433 to 25.535°. | |
| Limiting indices | -40<=h<=40, -40<=k<=40, -90<=l<=90 | |
| Reflections collected | 303957 | |
| Independent reflections | 30134 [R(int) = 0.0709] | |
| Completeness to theta | 25.242°/99.9% | |
| Absorption correction | Semi-empirical from equivalents | |
| Max. and min. transmission | 1.000 and 0.508 | |
| Refinement method | Full-matrix least-squares on F$^2$ | |
| Data / restraints / parameters | 30134 / 30 / 1429 | |
| Goodness-of-fit on F$^2$ | 1.082 | |
| Final R indices [I>2sigma(I)] | R1 = 0.0640, wR2 = 0.2086 | |
| R indices (all data) | R1 = 0.0698, wR2 = 0.2176 | |
| Extinction coefficient | 0.000033(2) | |
| Largest diff. peak and hole (e. Å$^{-3}$) | 1.69 and -1.03 | |

**Table S5** Crystallographic data of **5** – {$Mo_{132}$(3-Butenoic acid)}

| | | |
|---|---|---|
| Identification code | **5** | |
| Empirical formula | C120 H926 Mo132 N48 O736 S3 | |
| Formula weight | 27583.29 | |
| Temperature (K) | 150(2) | |
| Wavelength (Å) | 0.6889 | |
| Crystal system | Trigonal | |
| Space group | R-3 | |
| Unit cell dimensions | a = 32.4465(2) Å | $\alpha$= 90° |
| | b = 32.4465(2) Å | $\beta$= 90° |
| | c = 72.8870(5) Å | $\gamma$ = 120° |
| Volume (Å$^3$), Z | 66453.3(9), 3 | |
| Density (calculated) (mg/m$^3$) | 2.068 | |
| Absorption coefficient (mm$^{-1}$) | 1.916 | |
| F(000) | 40386 | |
| Crystal size (mm$^3$) | 0.100 x 0.100 x 0.030 | |
| θ range for data collection (°) | 1.463 to 25.537°. | |
| Limiting indices | -40<=h<=40, -40<=k<=40, -91<=l<=91 | |
| Reflections collected | 301757 | |
| Independent reflections | 30227 [R(int) = 0.0823] | |
| Completeness to theta | 25.242°/99.9% | |
| Absorption correction | Semi-empirical from equivalents | |
| Max. and min. transmission | 1.000 and 0.599 | |
| Refinement method | Full-matrix least-squares on F$^2$ | |
| Data / restraints / parameters | 30227 / 40 / 1465 | |
| Goodness-of-fit on F$^2$ | 1.066 | |
| Final R indices [I>2sigma(I)] | R1 = 0.0598, wR2 = 0.1943 | |
| R indices (all data) | R1 = 0.0661, wR2 = 0.2012 | |
| Extinction coefficient | 0.000024(3) | |
| Largest diff. peak and hole (e. Å$^{-3}$) | 1.79 and -1.16 | |

**(10) Characterization of 1 – {$Mo_{132}$(Acrylic acid)}**

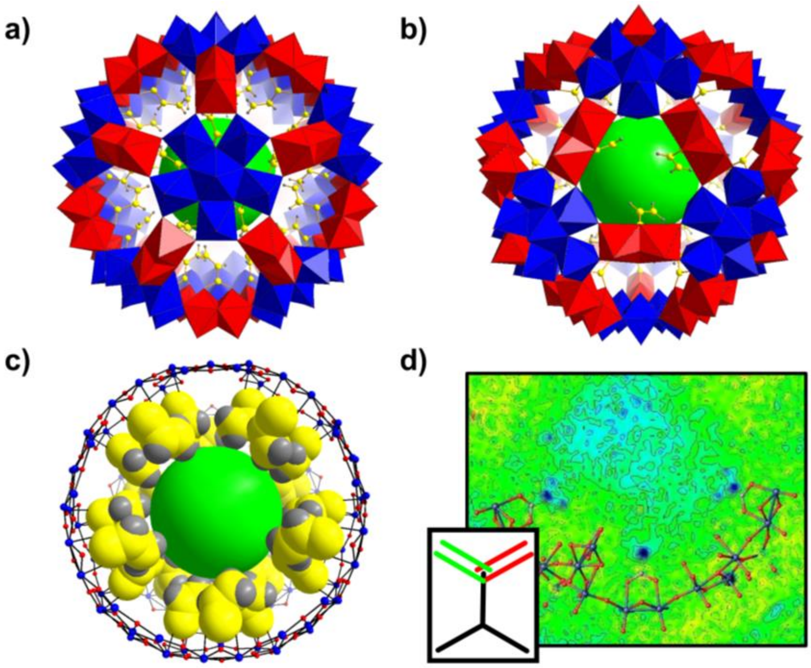


**Figure S4: a)** Polyhedral framework representation of **1** with ball-and-stick acrylic acid ligands, viewed along one of the 12 pentagonal {(Mo)$Mo_5$} units, **b)** polyhedral framework representation of **1** with ball-and-stick acrylic acid ligands, viewed along one of the 20 {$Mo_9O_9$} pores, **c)** ball-and stick framework representation of **1** with space-filling acrylic acid ligands, with one pentagonal unit and 5 {$Mo_2$} units and all non-coordinating oxygen atoms removed for clarity, **d)** electron density map of **1** with a partial section of the framework and ligand atoms shown, with emphasis of the two distinct orientations of the acrylic acid ligand, as a result of rotational isomerization, shown in the inset. For **a)-c)**, a green sphere has been added to fill the central cavity. Key: polyhedra – red = {$Mo_2$} linker-type units, blue = {(Mo)$Mo_5$} pentagonal-type units; atoms - red = oxygen, blue = molybdenum, yellow = carbon, grey = hydrogen; electron density – blue = high, and green = low electron density.

*(10.1) Thermogravimetric Analysis of 1*

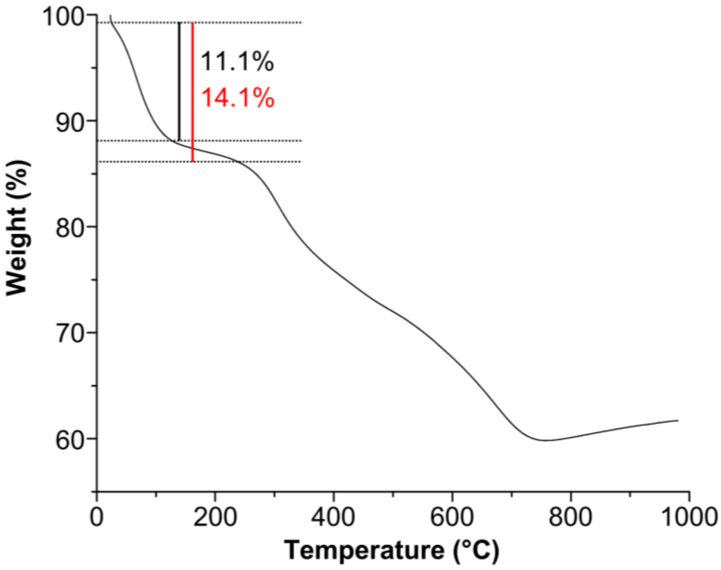


**Figure S5:** TGA of **1**. The percentage weight loss due to water loss between 50-225 °C is highlighted, representing approximately 180 solvent molecules.

*(10.2) NMR Study of 1*

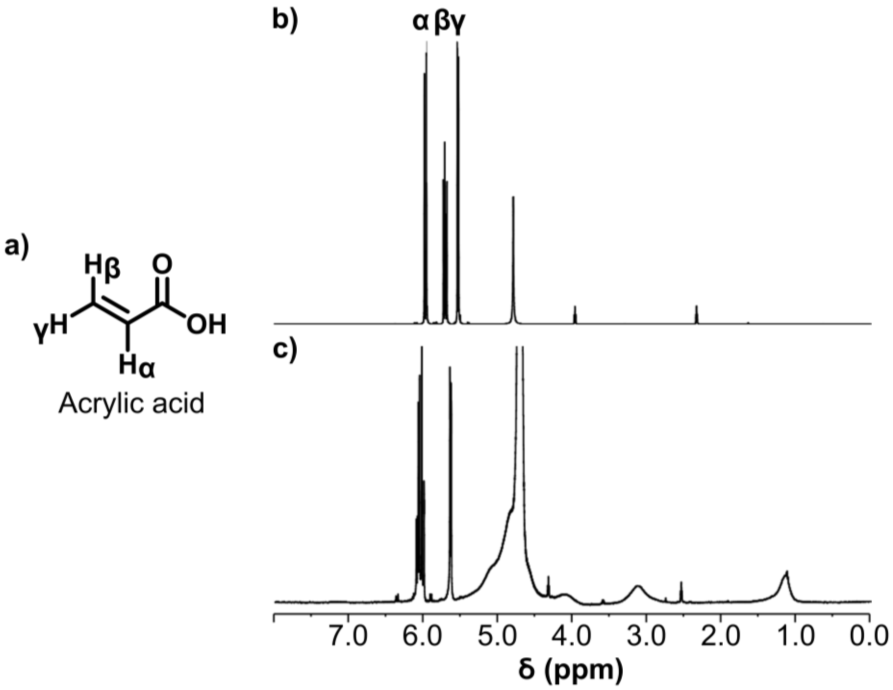


**Figure S6: a)** Structure of acrylic acid with labelled proton groups, **b)** $^1H$ NMR spectrum of acrylic acid in $D_2O$, and **c)** $^1H$ NMR spectrum of **1** in $D_2O$. The peaks arising from the solvated acrylic acid ligand species are highlighted: **α**

= 5.9 ppm, **β** = 5.7 ppm, and **γ** = 5.5 ppm. The encapsulated acrylic acid ligand species cannot be resolved due to overlapping signals between the water peak and the encapsulated species, at approximately 4.5-5.5 ppm. In addition, impurity peaks are present at 4.0 ppm and 2.4 ppm (for the free ligand), with related broad peaks at 3.2 ppm and 1.2 ppm. Using additional NMR techniques, presented in the SI, the impurity was determined to be the acrylic acid dimer, 3-(acryloyloxy)propanoic acid, which is known to form spontaneously in stored solutions of acrylic acid.[27]

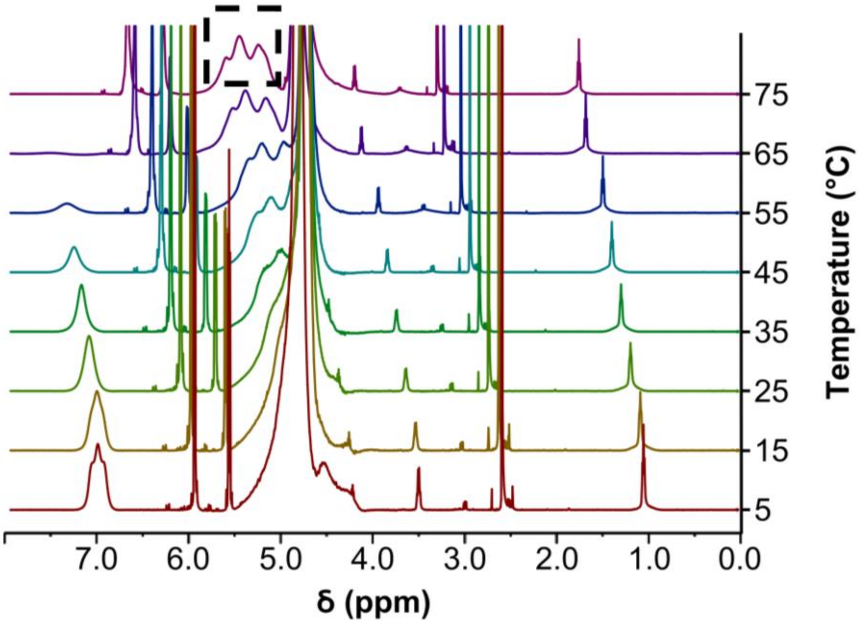


**Figure S7:** Variable-temperature (5-75 °C) $^1$H NMR spectrum of **1** in $D_2O$. The peaks previously 'hidden' by the water peak at room temperature measurements are effectively separated from this position and appear distinct from that of the water peak at temperatures greater than 45 °C (highlighted by the dashed box), between 5.5 ppm and 5.1 ppm.

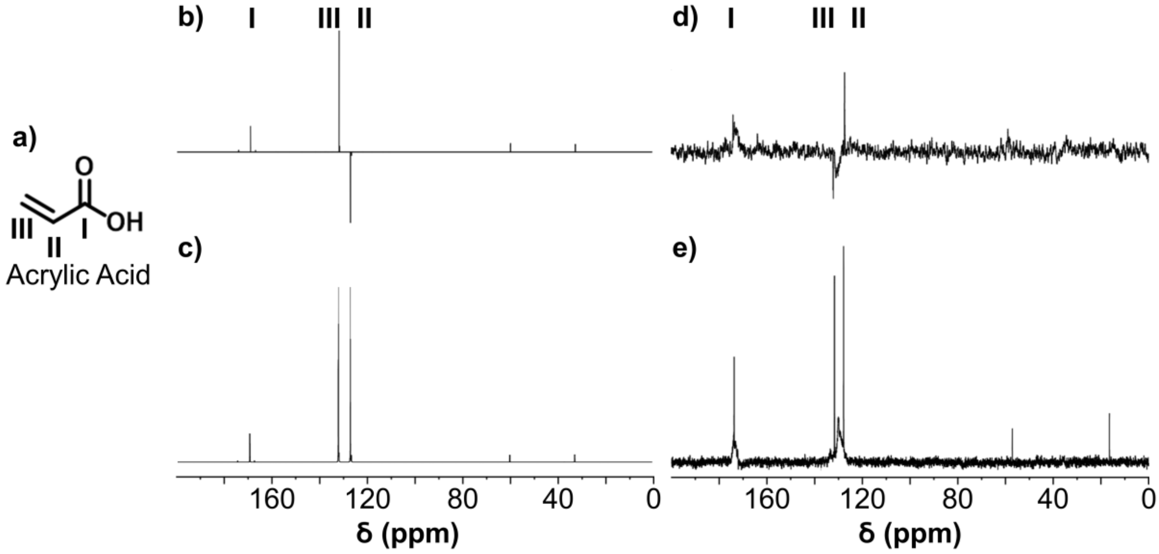


**Figure S8: a)** Assignment of acrylic acid for use in $^{13}$C NMR spectra labelling, **b)** $^{13}$C DEPTQ NMR spectrum of acrylic acid in $D_2O$, **c)** $^{13}$C NMR spectrum of acrylic acid in $D_2O$, **d)** $^{13}$C DEPTQ NMR spectrum of **1** in $D_2O$, and **e)** $^{13}$C NMR spectrum of **1** in $D_2O$. For the $^{13}$C DEPTQ NMR measurements signals arising due to quaternary carbon nuclei ($C_q$) and methylene ($CH_2$) carbon nuclei appear pointing upwards, whilst methine (CH) and methyl ($CH_3$) carbon nuclei appear pointing downwards. The peaks arising from the solvated acrylic acid ligand species are highlighted: **I** = 170 ppm (-**C**OO$^-$), **II** = 127 ppm (-**C**H), and **III** = 133 ppm (-**C**$H_2$). The encapsulated acrylic acid ligand species appear slightly shifted and broadened due to the same effects expressed in the $^1$H NMR spectra. In addition, impurity peaks are present at 61 ppm and 33 ppm.

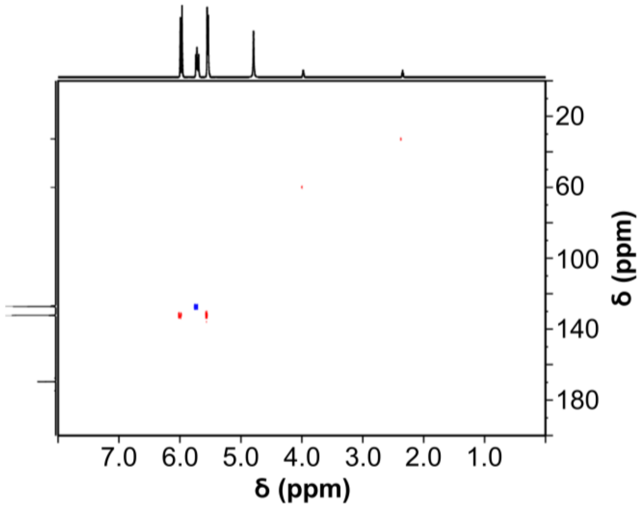


**Figure S9:** $^1$H-$^{13}$C multiplicity-edited HSQC 2D NMR spectrum of acrylic acid in $D_2O$. Here, the CH and $CH_3$ signals are highlighted as blue, whilst $CH_2$ signals are highlighted red. The impurity signals ($^1$H: 4.0 ppm and 2.4

ppm, $^{13}C$: 61 ppm and 33 ppm) are therefore assigned as $CH_2$ groups, confirming the presence of 3-(acryloyloxy)propanoic acid.

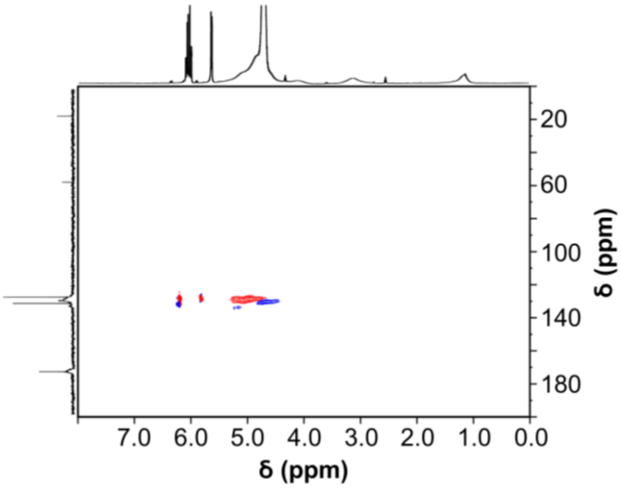


**Figure S10:** $^{1}H$-$^{13}C$ multiplicity-edited HSQC 2D NMR spectrum of **1** in $D_2O$. The broad signals hidden by the water peak, attributed to encapsulated acrylic acid ligands can be partially resolved using this technique.

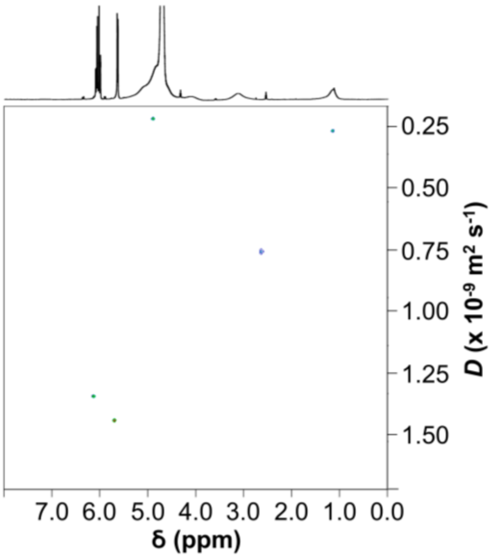


**Figure S11:** $^{1}H$ DOSY NMR spectrum of **1** in $D_2O$. The signals arising from the solvated acrylic acid ligand species (**αβγ**) and the encapsulated acrylic acid ligand species are highlighted. The solvated acrylic acid ligands have a diffusion coefficient of approximately 1.40 x $10^{-9}$ $m^2$ $s^{-1}$, whilst the encapsulated acrylic acid signals possess diffusion coefficients of approximately 2.50 x $10^{-10}$ $m^2$ $s^{-1}$. The large discrimination of the two values provides further evidence of their origins from species in two different domains.

## (11) Characterization of 2 – {$Mo_{132}$(Crotonic acid)}

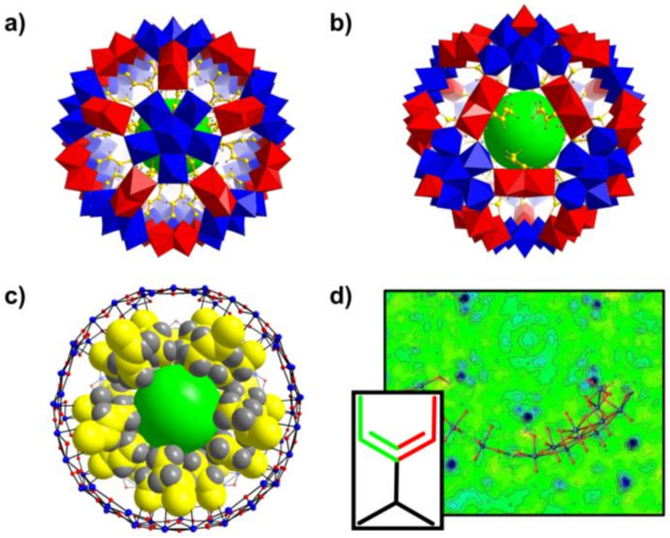


**Figure S12:** a) Polyhedral framework representation of **2** with ball-and-stick crotonic acid ligands, viewed along one of the 12 pentagonal {(Mo)$Mo_5$} units, b) polyhedral framework representation of **2** with ball-and-stick crotonic acid ligands, viewed along one of the 20 {$Mo_9O_9$} pores, c) ball-and stick framework representation of **2** with space-filling crotonic acid ligands, with one pentagonal unit and 5 {$Mo_2$} units and all non-coordinating oxygen atoms removed for clarity, d) electron density map of **2** with a partial section of the framework and ligand atoms shown, with emphasis of the two distinct orientations of the crotonic acid ligand, as a result of rotational isomerization, shown in the inset. For **a**)-**c**), a green sphere has been added to fill the central cavity. Key: polyhedra – red = {$Mo_2$} linker-type units, blue = {(Mo)$Mo_5$} pentagonal-type units; atoms - red = oxygen, blue = molybdenum, yellow = carbon, grey = hydrogen; electron density – blue = high, and green = low electron density.

### *(11.1) Thermogravimetric analysis of 2*

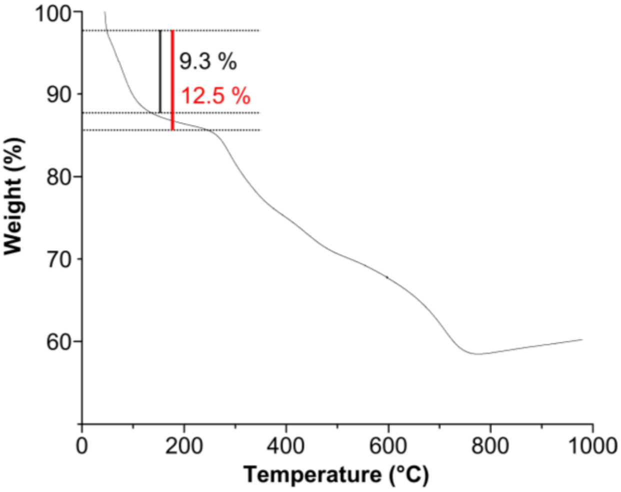


**Figure S13:** TGA of **2**. The percentage weight loss due to water loss between 50-225 °C is highlighted, representing approximately 160 solvent molecules.

***(11.2) NMR Study of 2***

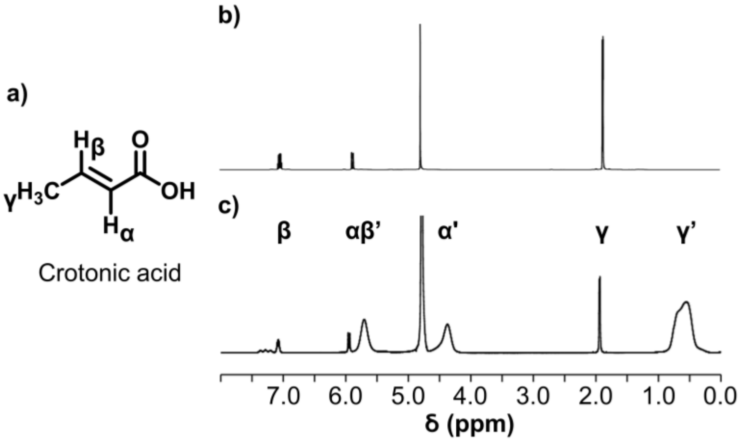


**Figure S14: a)** Structure of crotonic acid with labelled proton groups **b)** $^{1}$H NMR spectrum of crotonic acid in $D_2O$, and **c)** $^{1}$H NMR spectrum of **2** in $D_2O$. The peaks arising from the solvated crotonic acid ligand species are highlighted: **α** = 5.9 ppm, **β** = 7.0 ppm, and **γ** = 1.9 ppm. The encapsulated crotonic acid ligand species peaks appear significantly broadened and upfield shifted due to the coordination of the species to the negatively charged $\{Mo_{132}\}$, framework in addition to the confined nature of the ligand environment. The resultant peaks appear at **α'** = 4.4 ppm, **β'** = 5.7 ppm, and **γ'** = 0.7 ppm, with shifts of **α-α'** = -1.5 ppm, **β-β'** = -1.3 ppm, and **γ-γ'** = -1.2 ppm.

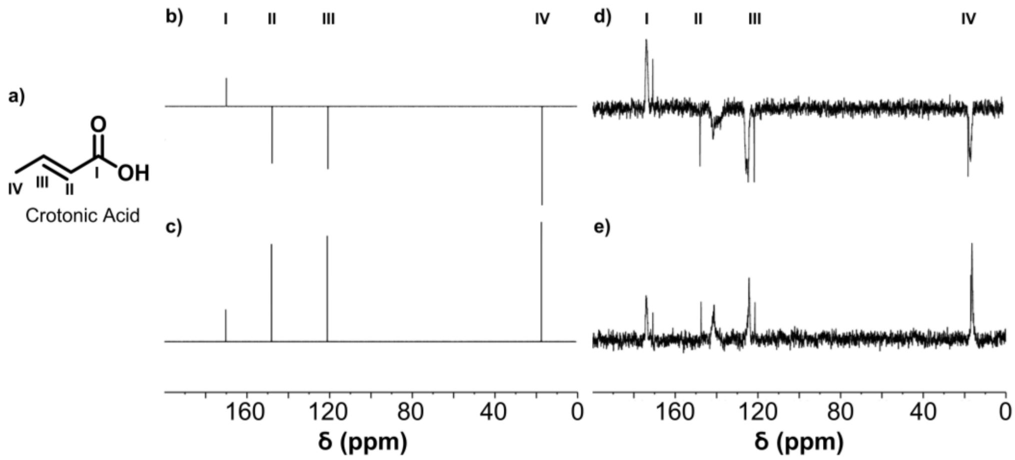


**Figure S15: a)** Assignment of crotonic acid for use in $^{13}$C NMR spectra labelling, **b)** $^{13}$C DEPTQ NMR spectrum of crotonic acid in $D_2O$, **c)** $^{13}$C NMR spectrum of crotonic acid in $D_2O$, d**)** $^{13}$C DEPTQ NMR spectrum of **2** in $D_2O$, and **e**) $^{13}$C NMR spectrum of **2** in $D_2O$. For the $^{13}$C DEPTQ NMR measurements signals arising due to quaternary carbon nuclei ($C_q$) and methylene ($CH_2$) carbon nuclei appear pointing upwards, whilst methine (CH) and methyl ($CH_3$) carbon nuclei appear pointing downwards. The peaks arising from the solvated crotonic acid ligand species are highlighted: **I** = 171 ppm (-**C**$OO^-$), **II** = 148 ppm (-**C**H), **III** = 121 ppm (-**C**H), and **IV** = 17 ppm (-**C**$H_3$). The

encapsulated crotonic acid ligand species appear slightly shifted and broadened due to the same effects expressed in the $^{1}H$ NMR spectra.

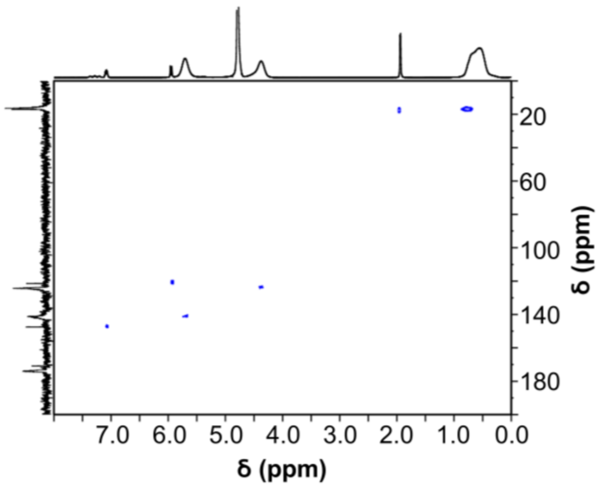


**Figure S16:** $^{1}H$-$^{13}C$ multiplicity-edited HSQC 2D NMR spectrum of **2** in $D_2O$. The CH and $CH_3$ signals are highlighted as blue. The spectrum confirms the correlation between peaks of the free crotonic acid and their related encapsulated peak signals, in both the $^{13}C$ and $^{1}H$ spectra.

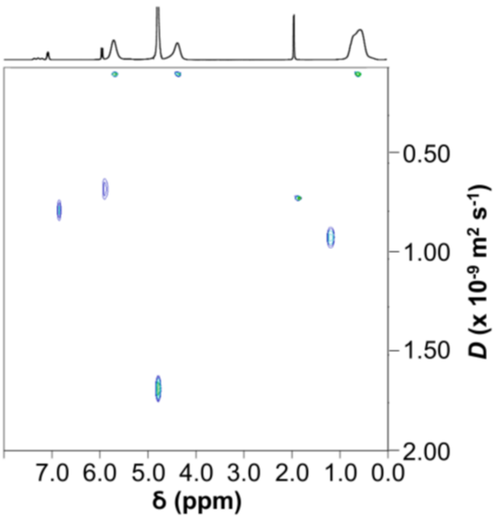


**Figure S17:** $^{1}H$ DOSY NMR spectrum of **2** in $D_2O$. The signals arising from the solvated crotonic acid ligand species (**αβγ**) and the encapsulated crotonic acid ligand species (**α'β'γ'**) are highlighted. The solvated crotonic acid ligands have a diffusion coefficient of approximately 7.62 x $10^{-10}$ $m^2$ $s^{-1}$, whilst the encapsulated crotonic acid signals possess diffusion coefficients of approximately 1.15 x $10^{-10}$ $m^2$ $s^{-1}$. The large discrimination of the two values provides further evidence of their origins from species in two different domains. An additional peak arising from water has a diffusion coefficient of 1.75 x $10^{-9}$ $m^2$ $s^{-1}$. Finally, an impurity peak at 1.1 ppm has a similar diffusion coefficient to the free crotonic acid species, 9.56 x $10^{-10}$ $m^2$ $s^{-1}$.

## (12) Characterization of 3 – {$Mo_{132}$(Methacrylic acid)}

### *(12.1) Thermogravimetric Analysis of 3*

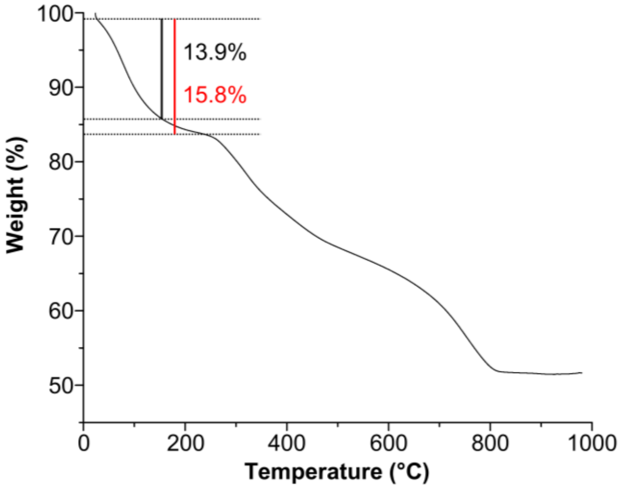


**Figure S18:** TGA of **3**. The percentage weight loss due to water loss between 50-225 °C is highlighted, representing approximately 210 solvent molecules.

### *(12.2) NMR Study of 3*

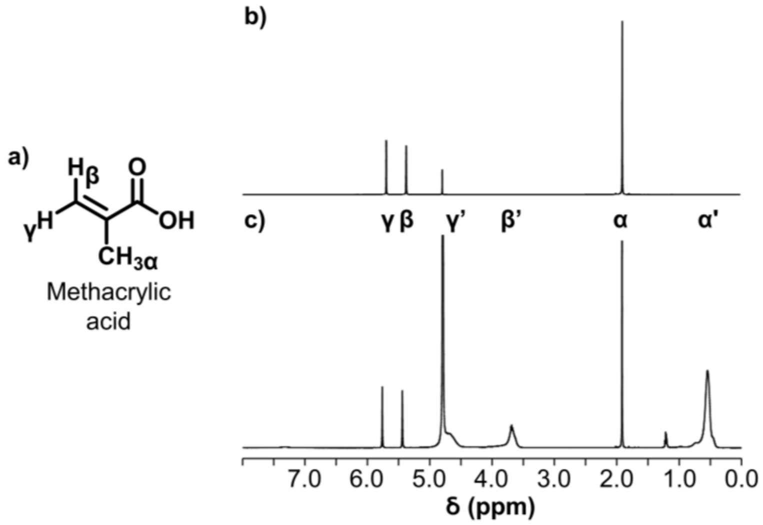


**Figure S19: a)** Structure of methacrylic acid with labelled proton groups, **b)** $^{1}$H NMR spectrum of methacrylic acid in $D_2O$, and **c)** $^{1}$H NMR Spectrum of **3** in $D_2O$. The peaks arising from the solvated methacrylic acid ligand species are highlighted: **α** = 1.9 ppm, **β** = 5.4 ppm, and **γ** = 5.7 ppm. The encapsulated methacrylic acid ligand species peaks appear significantly broadened and upfield shifted due to the coordination of the species to the negatively charged

$\{Mo_{132}\}$ framework, in addition to the confined nature of the ligand environment. The resultant peaks appear at **α'** = 0.6 ppm, **β'** = 3.7 ppm, and **γ'** = 4.7 ppm, with shifts of **α-α'** = -1.3 ppm, **β-β'** = -1.7 ppm, and **γ-γ'** = -1.0 ppm.

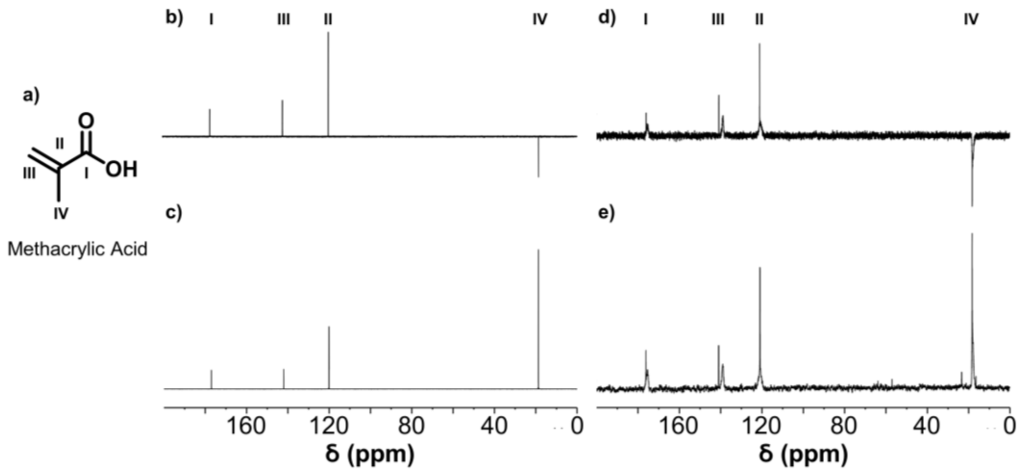


**Figure S20: a)** Assignment of methacrylic acid for use in $^{13}C$ NMR spectra labelling, **b)** $^{13}C$ DEPTQ NMR spectrum of methacrylic acid in $D_2O$, **c)** $^{13}C$ NMR spectrum of methacrylic acid in $D_2O$, **d)** $^{13}C$ DEPTQ NMR spectrum of **3** in $D_2O$, and **e)** $^{13}C$ NMR spectrum of **3** in $D_2O$. For the $^{13}C$ DEPTQ NMR measurements signals arising due to quaternary carbon nuclei ($C_q$) and methylene ($CH_2$) carbon nuclei appear pointing upwards, whilst methine (CH) and methyl ($CH_3$) carbon nuclei appear pointing downwards. The peaks arising from the solvated methacrylic acid ligand species are highlighted: **I** = 177 ppm (-**C**$OO^-$), **II** = 121 ppm (-**C**-), **III** = 142 ppm (-**C**$H_2$), and **IV** = 19 ppm (-**C**$H_3$). The encapsulated methacrylic acid ligand species peaks appear slightly shifted and broadened due to the same effects expressed in the $^1H$ NMR spectra.

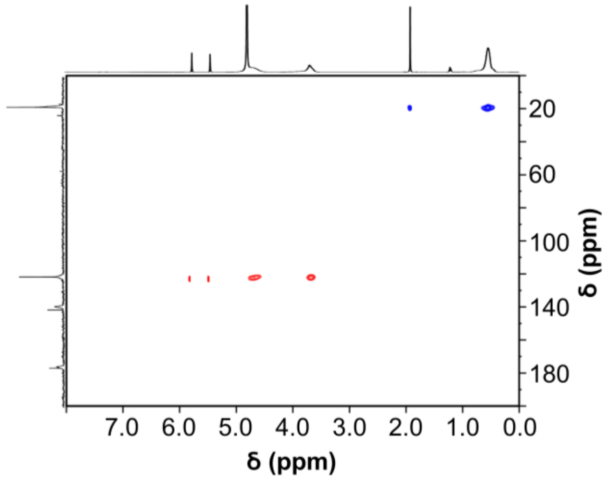


**Figure S21:** $^1H$-$^{13}C$ multiplicity-edited HSQC 2D NMR spectrum of **3** in $D_2O$. Here, $CH_3$ signals are highlighted in blue, whilst $CH_2$ signals are highlighted in red. The spectrum confirms the correlation between peaks originating from the free methacrylic acid and the related encapsulated peak signals, in both the $^{13}C$ and $^1H$ spectra.

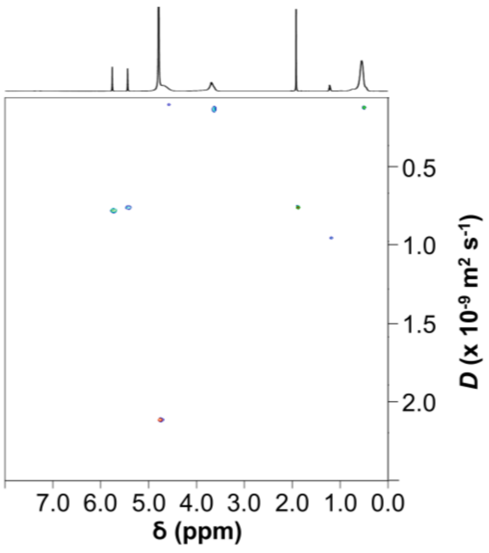


**Figure S22:** $^{1}$H DOSY NMR spectrum of **3** in $D_2O$. The signals arising from the solvated methacrylic acid ligand species (**αβγ**) and the encapsulated methacrylic acid ligand species (**α'β'γ'**) are highlighted. The solvated methacrylic acid ligands have a diffusion coefficient of approximately 7.58 x $10^{-10}$ $m^2$ $s^{-1}$, whilst the encapsulated methacrylic acid signals possess diffusion coefficients of approximately 1.14 x $10^{-10}$ $m^2$ $s^{-1}$. The large discrimination of the two values provides further evidence of their origins from species in two different domains. An additional peak arising from water has a diffusion coefficient of 2.11 x $10^{-9}$ $m^2$ $s^{-1}$. Finally, an impurity peak at 1.1 ppm has a similar diffusion coefficient to the free methacrylic acid species, 9.49 x $10^{-10}$ $m^2$ $s^{-1}$.

### (13) Characterization of 4 – {$Mo_{132}$(Tiglic acid)}

#### *(13.1) Thermogravimetric Analysis of 4*

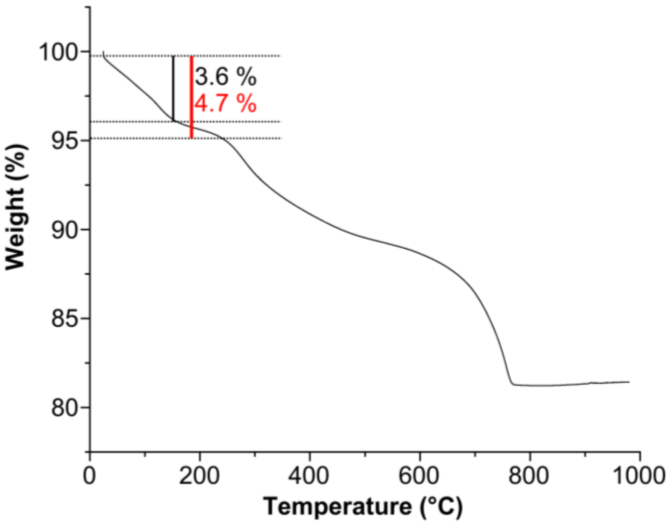

**Figure S23:** TGA of **4**. The percentage weight loss due to water loss between 50-225 °C is highlighted, representing approximately 80 solvent molecules.

***(13.2) NMR Study of 4***

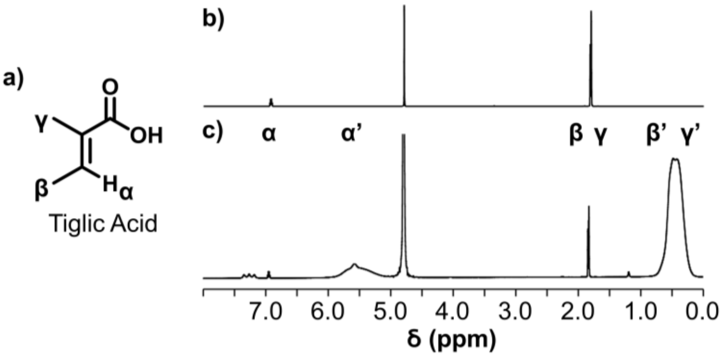


**Figure S24:** a) Structure of tiglic acid with labelled proton groups, b) $^1H$ NMR spectrum of tiglic acid in $D_2O$, and c) $^1H$ NMR spectrum of **4** in $D_2O$. The peaks arising from the solvated tiglic acid ligand species are highlighted: **α** = 6.9 ppm, and **β** and **γ** = 1.8 ppm. The encapsulated tiglic acid ligand species peaks appear significantly broadened and upfield shifted due to the coordination of the species to the negatively charged {$Mo_{132}$} framework, in addition to the confined nature of the ligand environment. The resultant peaks appear at **α'** = 5.6 ppm, and **β'** and **γ'** = 0.4 ppm, with shifts of **α-α'** = -1.3 ppm, and **β-β'** and **γ-γ'** = -1.4 ppm.

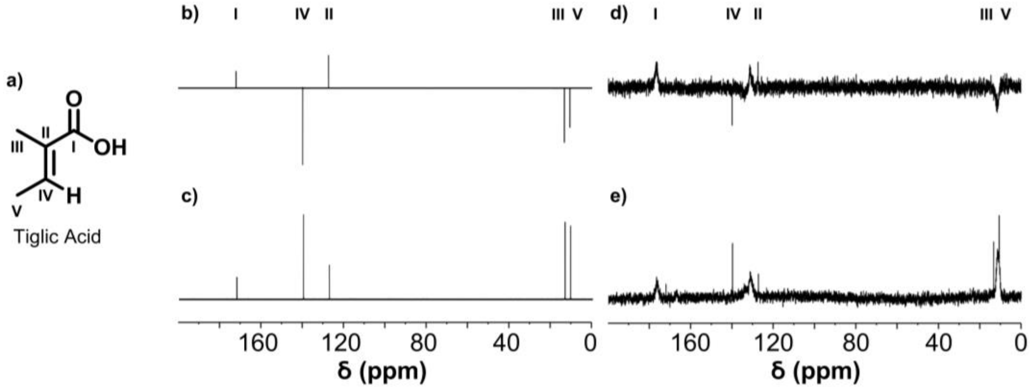


**Figure S25: a)** Assignment of tiglic acid for use in $^{13}C$ NMR spectra labelling, **b)** $^{13}C$ DEPTQ NMR spectrum of tiglic acid in $D_2O$, **c)** $^{13}C$ NMR spectrum of tiglic acid in $D_2O$, **d)** $^{13}C$ DEPTQ NMR spectrum of **4** in $D_2O$, and **e)** $^{13}C$ NMR spectrum of **4** in $D_2O$. For the $^{13}C$ DEPTQ NMR measurements signals arising due to quaternary carbon nuclei ($C_q$) and methylene ($CH_2$) carbon nuclei appear pointing upwards, whilst methine (CH) and methyl ($CH_3$) carbon nuclei appear pointing downwards. The peaks arising from the solvated tiglic acid ligand species are highlighted: **I** = 176 ppm (-**C**$OO^-$), **II** = 127 ppm (-**C**-), **III/V** = 10/13 ppm (-**C**$H_3$), and **IV** = 140 ppm (-**C**H). The encapsulated tiglic acid ligand species peaks appear slightly shifted and broadened due to the same effects expressed in the $^1H$ NMR spectra.

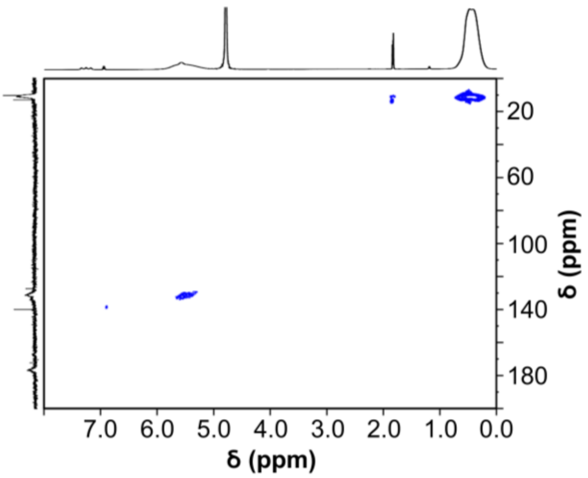


**Figure S26:** $^1H$-$^{13}C$ multiplicity-edited HSQC 2D NMR spectrum of **4** in $D_2O$. Here, CH and $CH_3$ signals are highlighted in blue. The spectrum confirms the correlation between peaks originating from the free tiglic acid and the related encapsulated peak signals, in both the $^{13}C$ and $^1H$ spectra.

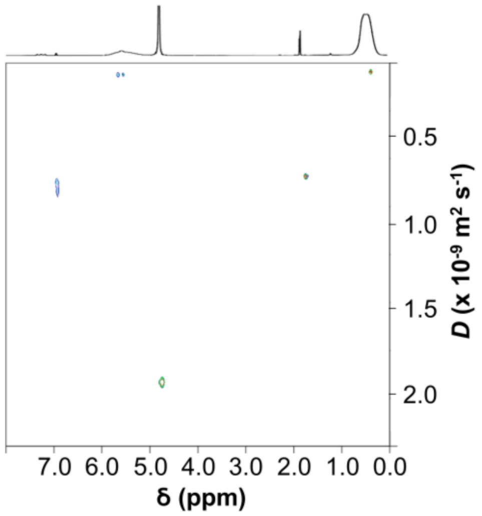


**Figure S27:** $^1H$ DOSY NMR spectrum of **4** in $D_2O$. The signals arising from the solvated tiglic acid ligand species (**αβγ**) and the encapsulated tiglic acid ligand species (**α'β'γ'**) are highlighted. The solvated tiglic acid ligands have a diffusion coefficient of approximately 7.51 x $10^{-10}$ $m^2$ $s^{-1}$, whilst the encapsulated tiglic acid signals possess diffusion coefficients of approximately 1.21 x $10^{-10}$ $m^2$ $s^{-1}$. The large discrimination of the two values provides further evidence of their origins from species in two different domains. An additional peak arising from water has a diffusion coefficient of 1.93 x $10^{-9}$ $m^2$ $s^{-1}$.

**(14) Characterization of 5 – {$Mo_{132}$(3-Butenoic acid)}**

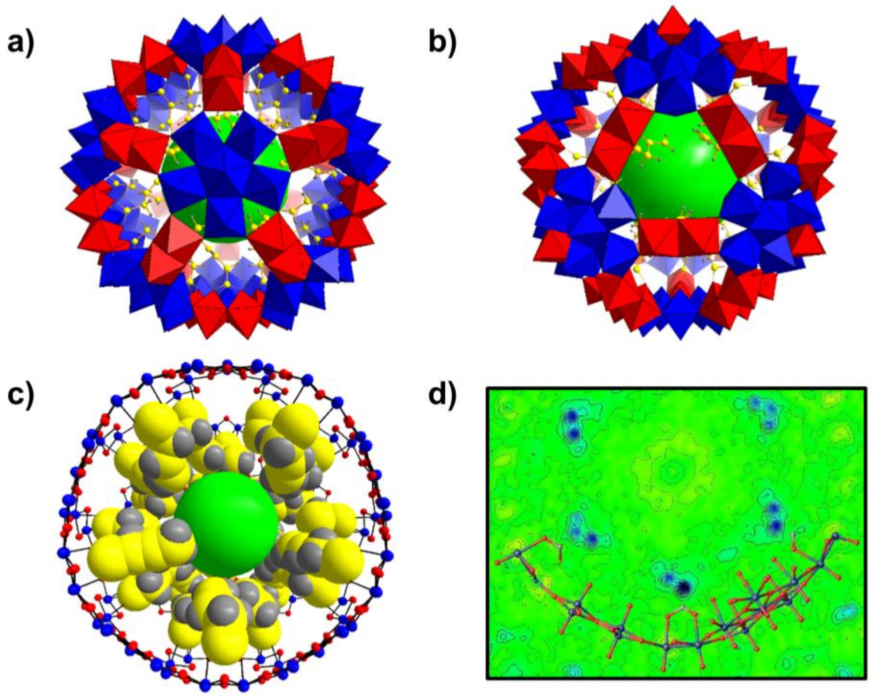


**Figure S28: a)** Polyhedral framework representation of **5** with ball-and-stick 3-butenoic acid ligands, viewed along one of the 12 pentagonal {(Mo)$Mo_5$} units, **b)** polyhedral framework representation of **5** with ball-and-stick 3-butenoic acid ligands, viewed along one of the 20 {$Mo_9O_9$} pores, **c)** ball-and stick framework representation of **5** with space-filling 3-butenoic acid ligands, with one pentagonal unit and 5 {$Mo_2$} units and all non-coordinating oxygen atoms removed for clarity, **d)** electron density map of **5** with a partial section of the framework and ligand atoms shown. For **a)**-**c)**, a green sphere has been added to fill the central cavity. Key: polyhedra – red = {$Mo_2$} linker-type units, blue = {(Mo)$Mo_5$} pentagonal-type units; atoms - red = oxygen, blue = molybdenum, yellow = carbon, grey = hydrogen; electron density – blue = high, and green = low electron density.

*(14.1) Thermogravimetric Analysis of 5*

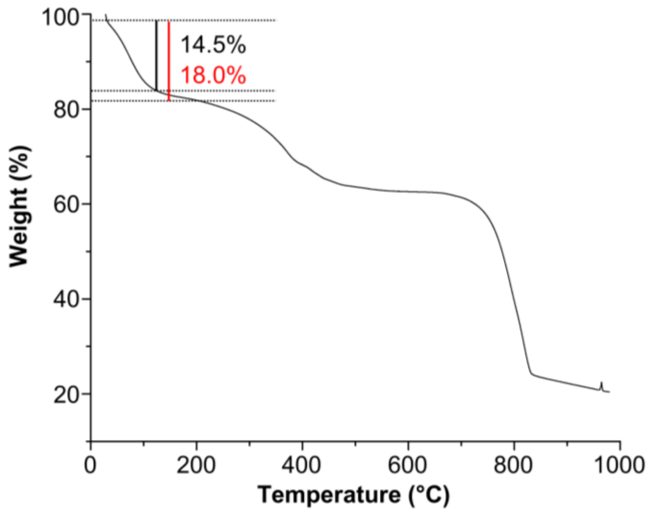


**Figure S29:** TGA of **5**. The percentage weight loss due to water loss between 50-225 °C is highlighted, representing approximately 220 solvent molecules.

*(14.2) NMR Study of 5*

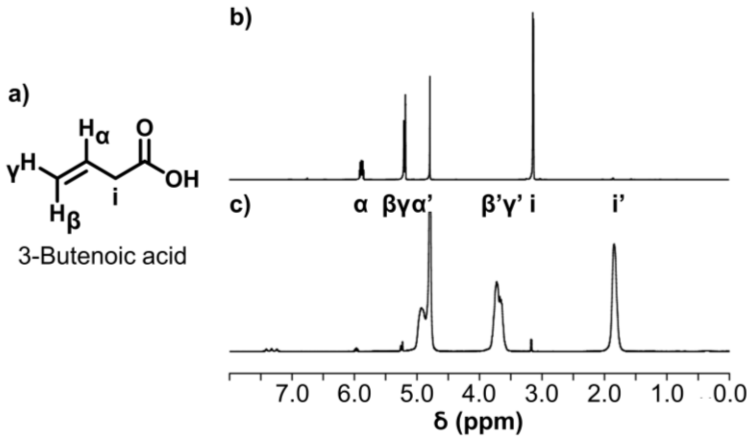


**Figure S30: a)** Structure of 3-butenoic acid with labelled proton groups, **b)** $^{1}$H NMR spectrum of 3-butenoic acid in $D_2O$, and **c)** $^{1}$H NMR spectrum of **5** in $D_2O$. The peaks arising from the solvated 3-butenoic acid ligand species are highlighted: **α** = 6.0 ppm, **β/γ** = 5.3 ppm, and **i** = 3.2 ppm. The encapsulated 3-butenoic acid ligand species peaks appear significantly broadened and upfield shifted due to the coordination of the species to the negatively charged $\{Mo_{132}\}$ framework, in addition to the confined nature of the ligand environment. The resultant peaks appear at, **α'** = 4.9 ppm, **β'/γ'** = 3.7 ppm, and **i'** = 1.8 ppm, with shifts of **α-α'**= -1.1 ppm, **β-β'/γ-γ'** = -1.6 ppm, and **i-i'** = -1.4 ppm.

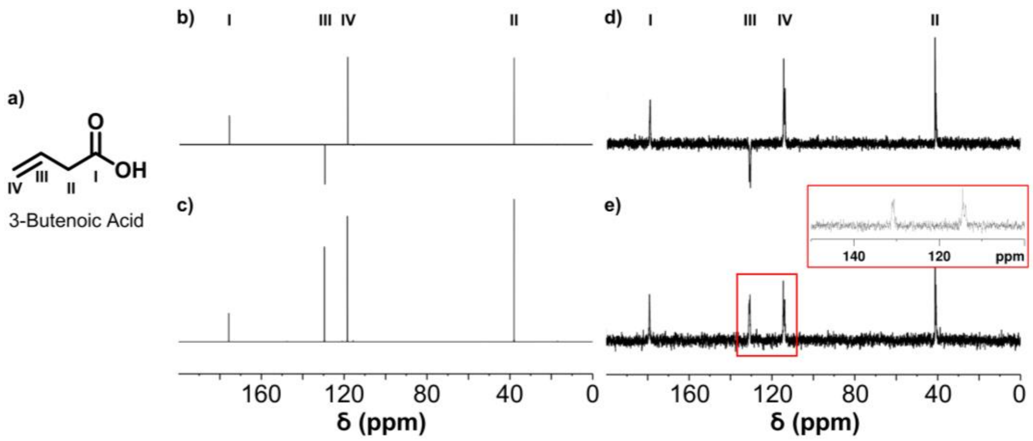


**Figure S31: a)** Assignment of 3-butenoic acid for use in $^{13}$C NMR spectra labelling, **b)** $^{13}$C DEPTQ NMR spectrum of 3-butenoic acid in $D_2O$, **c)** $^{13}$C NMR spectrum of 3-butenoic acid in $D_2O$, **d)** $^{13}$C DEPTQ NMR spectrum of **5** in $D_2O$, and **e**) $^{13}$C NMR spectrum of **5** in $D_2O$, with expanded section showing broadened resonances relating to ligand coordination shown in the inset. For the $^{13}$C DEPTQ NMR measurements signals arising due to quaternary carbon nuclei ($C_q$) and methylene ($CH_2$) carbon nuclei appear pointing upwards, whilst methine (CH) and methyl ($CH_3$) carbon nuclei appear pointing downwards. The peaks arising from the solvated 3-butenoic acid ligand species are highlighted: **I** = 179 ppm (-**C**OO$^-$), **II** = 40 ppm (-**C**H), **III** = 130 ppm (-**C**$H_2$), and **IV** = 114 ppm (-**C**$H_2$). The encapsulated 3-butenoic ligand species appear slightly shifted and broadened due to the same effects expressed in the $^1$H NMR spectra.

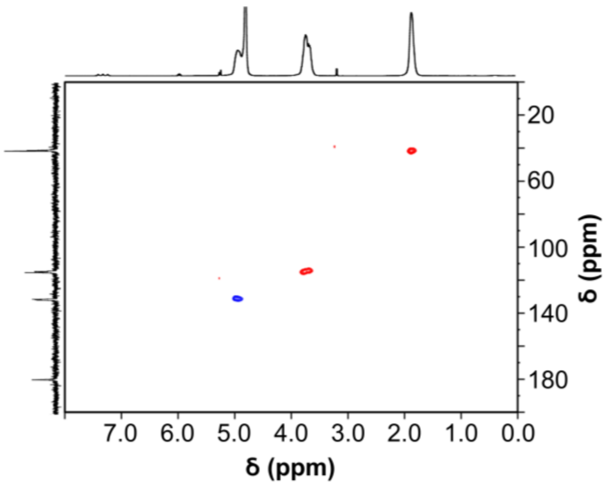


**Figure S32:** $^1$H-$^{13}$C multiplicity-edited HSQC 2D NMR spectrum of **5** in $D_2O$. Here, CH and $CH_3$ signals are highlighted in blue, whilst $CH_2$ signals are highlighted in red. The spectrum confirms the correlation between peaks originating from the free 3-butenoic acid and the related encapsulated peak signals, in both the $^{13}$C and $^1$H spectra.

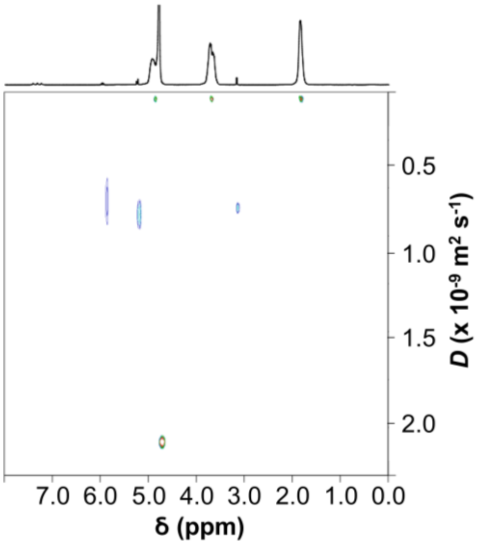


**Figure S33:** $^1$H DOSY NMR spectrum of **5** in $D_2O$. The signals arising from the solvated 3-butenoic acid ligand species (**αβγi**) and the encapsulated 3-butenoic acid ligand species (**α'β'γ'i'**) are highlighted. The solvated 3-butenoic acid ligands have a diffusion coefficient of approximately 7.00 x $10^{-10}$ $m^2$ $s^{-1}$, whilst the encapsulated 3-butenoic acid signals possess diffusion coefficients of approximately 1.10 x $10^{-10}$ $m^2$ $s^{-1}$. The large discrimination of the two values provides further evidence of their origins from species in two different domains.

The hydrodynamic radii of both the encapsulated and free 3-butenoic acid ligand species was estimated by application of the Stokes-Einstein equation (**Equation 1**):

$$r = \frac{kT}{6\pi\eta D}$$

Where, $r$ = van der Waals radius (m), $k$ = Boltzmann constant (1.380 x $10^{-23}$ J $K^{-1}$), $T$ = temperature (300 K), $\eta$ = solvent viscosity (0.000853 Pa s), and $D$ = diffusion coefficient ($m^2$ $s^{-1}$). The hydrodynamic radii were determined to be ~22 Å ($D$ = 0.000000000115 $m^2$ $s^{-1}$) and ~4 Å ($D$ = 0.000000000740 $m^2$ $s^{-1}$) for the broad encapsulated peaks and the sharp free peaks, respectively.

## (15) Characterization of 6 – {$Mo_{132}$(4-Pentenoic acid)}

### *(15.1) Thermogravimetric Analysis of 6*

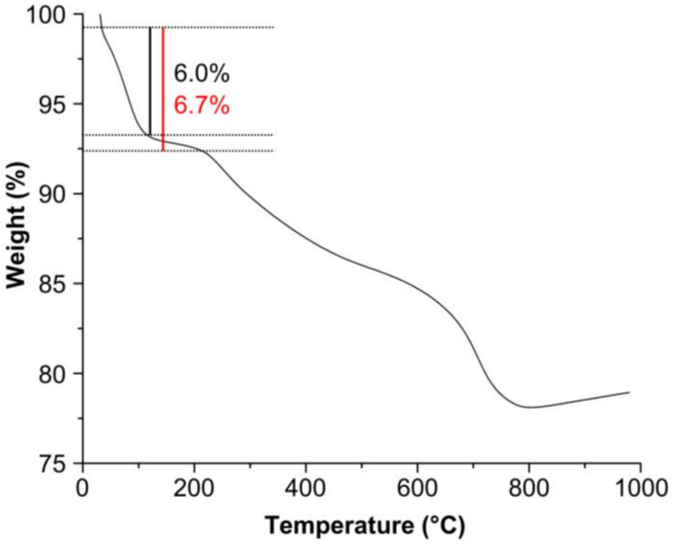


**Figure S34:** TGA of **6**. The percentage weight loss due to water loss between 50-225 °C is highlighted, representing approximately 90 solvent molecules.

### *(15.2) NMR Study of 6*

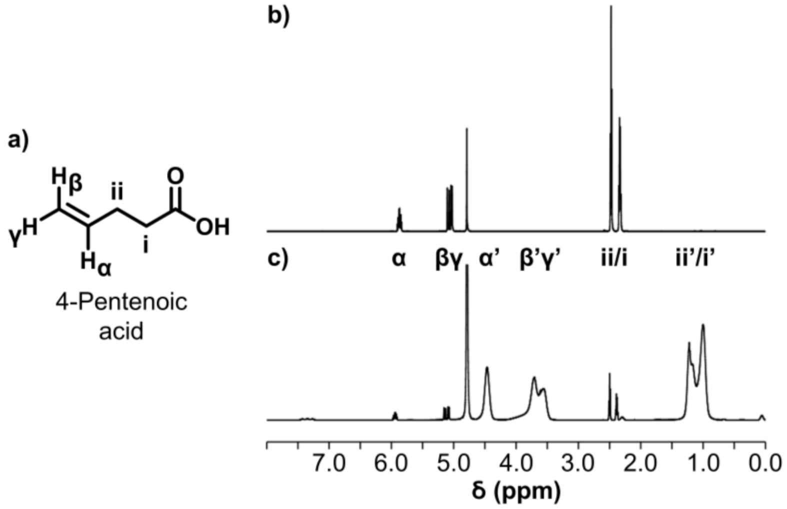


**Figure S35: a)** Structure of 4-pentenoic acid with labelled proton groups, **b)** $^{1}H$ NMR spectrum of 4-pentenoic acid in $D_2O$, and **c)** $^{1}H$ NMR spectrum of **6** in $D_2O$. The peaks arising from the solvated 4-pentenoic acid ligand species are highlighted: **α** = 6.0 ppm, **β/γ** = 5.1 ppm, and **i/ii** = 2.4 ppm. The encapsulated 4-pentenoic acid ligand species peaks appear significantly broadened and upfield shifted due to the coordination of the species to the negatively

charged {$Mo_{132}$} framework, in addition to the confined nature of the ligand environment. The resultant peaks appear at **α'** = 4.5 ppm, **β'/γ'** = 3.6-3.7 ppm, and **i'/ii'** = 1.0-1.2 ppm, with shifts of **α-α'** = -1.5 ppm, **β-β'/γ-γ'** = -1.5 ppm, and **i-i'/ii-ii'** = -1.2 ppm.

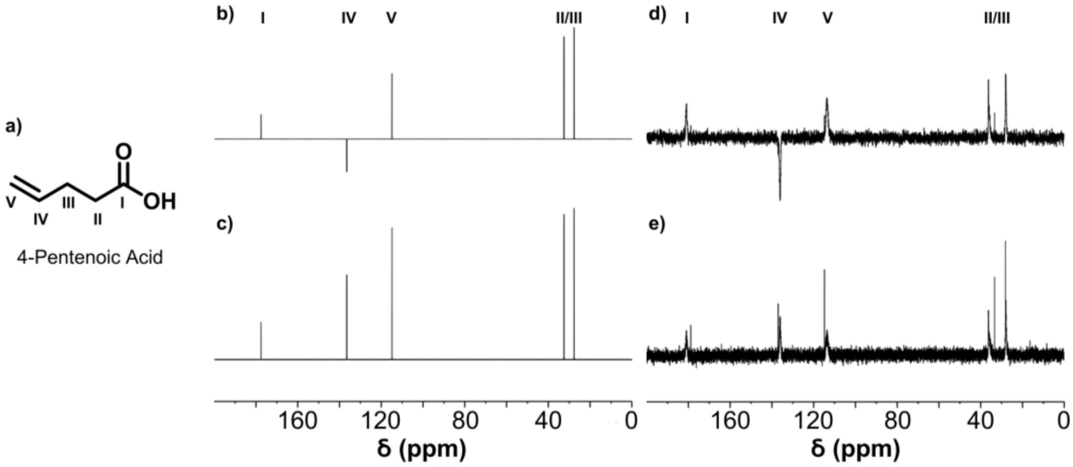


**Figure S36: a)** Assignment of 4-pentenoic acid for use in $^{13}$C NMR spectra labelling, **b)** $^{13}$C DEPTQ NMR spectrum of 4-pentenoic acid in $D_2O$, **c)** $^{13}$C NMR spectrum of 4-pentenoic acid in $D_2O$, **d)** $^{13}$C DEPTQ NMR spectrum of **6** in $D_2O$, and **e)** $^{13}$C NMR spectrum of **6** in $D_2O$. For the $^{13}$C DEPTQ NMR measurements signals arising due to quaternary carbon nuclei ($C_q$) and methylene ($CH_2$) carbon nuclei appear pointing upwards, whilst methine (CH) and methyl ($CH_3$) carbon nuclei appear pointing downwards. The peaks arising from the solvated 4-pentenoic ligand species are highlighted: **I** = 178 ppm (-**C**$OO^-$), **II** = 33 ppm (-**C**$H_2$), **III** = 28 ppm (-**C**$H_2$), **IV** = 137 ppm (-**C**H), and **V** = 115 ppm (-**C**$H_2$). The encapsulated 4-pentenoic acid ligand species appear slightly shifted and broadened due to the same effects expressed in the $^1$H NMR spectra.

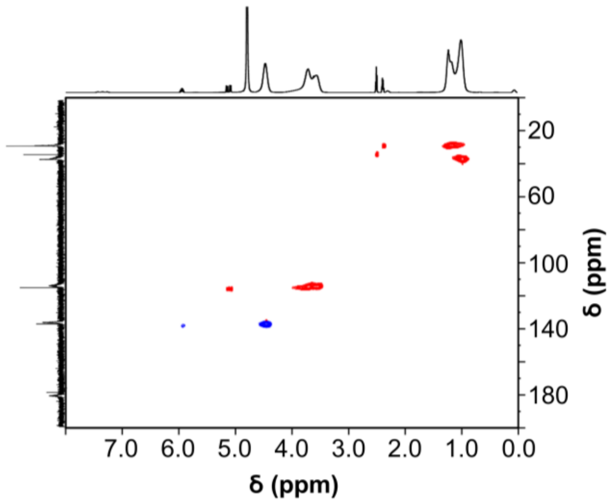


**Figure S37:** $^1$H-$^{13}$C multiplicity-edited HSQC 2D NMR spectrum of **6** in $D_2O$. Here, CH and $CH_3$ signals are highlighted in blue, whilst $CH_2$ signals are highlighted in red. The spectrum confirms the correlation between peaks originating from the free 4-pentenoic acid and the related encapsulated peak signals, in both the $^{13}$C and $^1$H spectra.

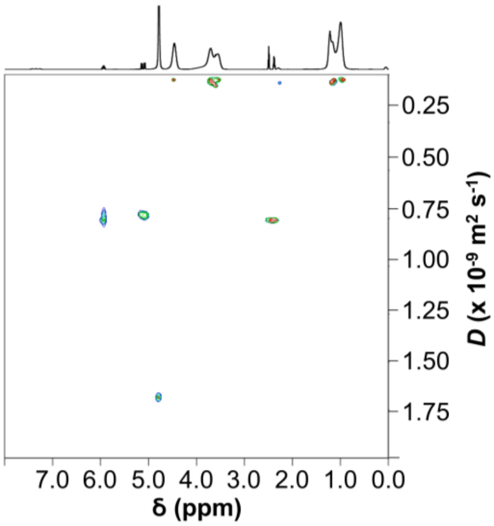


**Figure S38:** $^1$H DOSY NMR spectrum of **6** in $D_2O$. The signals arising from the solvated 4-pentenoic acid ligand species (**αβγi ii**) and the encapsulated 4-pentenoic acid ligand species (**α'β'γ'i'ii'**) are highlighted. The solvated 4-pentenoic acid ligands have a diffusion coefficient of approximately 7.77 x $10^{-10}$ $m^2$ $s^{-1}$, whilst the encapsulated 4-pentenoic acid signals possess diffusion coefficients of approximately 1.14 x $10^{-10}$ $m^2$ $s^{-1}$. The large discrimination of the two values provides further evidence of their origins from species in two different domains. An additional peak arising from water has a diffusion coefficient of 1.66 x $10^{-9}$ $m^2$ $s^{-1}$.

**(16) Characterization of 7 – {$Mo_{132}$(5-Hexenoic Acid)}**

***(16.1) Thermogravimetric Analysis of 7***

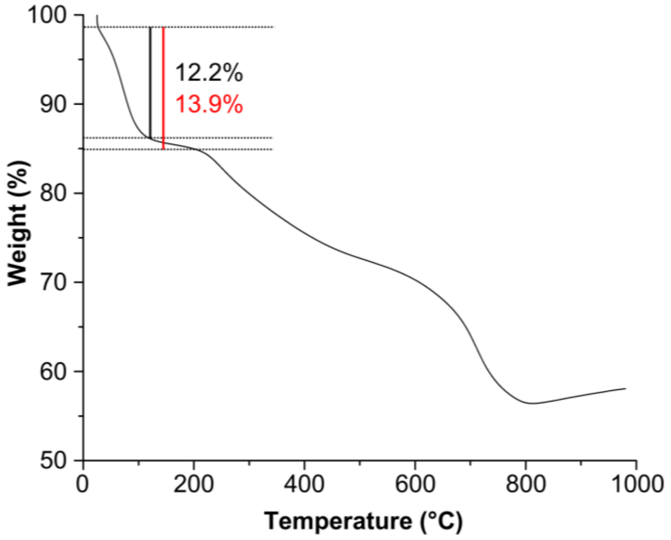


**Figure S39**: TGA of **7**. The percentage weight loss due to water loss between 50-225 °C is highlighted, representing approximately 200 solvent molecules.

*(16.2) NMR Study of 7*

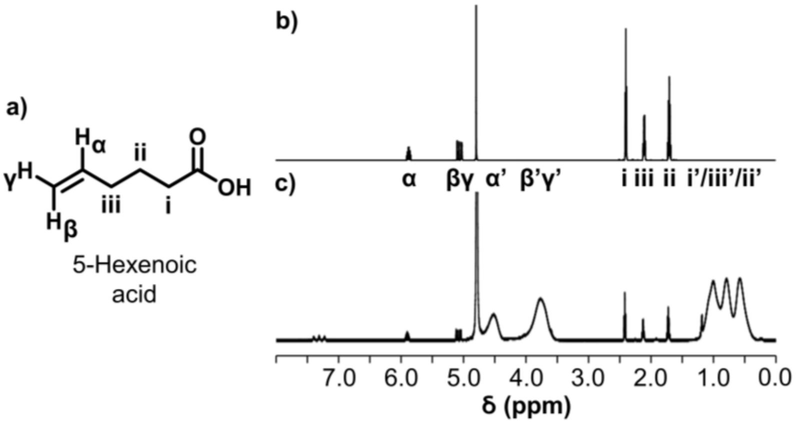


**Figure S40: a)** Structure of 5-hexenoic acid with labelled proton groups, **b)** $^{1}$H NMR spectrum of 5-hexenoic acid in $D_2O$, and **c)** $^{1}$H NMR spectrum of **7** in $D_2O$. The peaks arising from the solvated 5-hexenoic acid ligand species are highlighted: **α** = 5.9 ppm, **β/γ** = 5.1 ppm, **i** = 2.4 ppm, **ii** = 1.7 ppm, and **iii** = 2.1 ppm. The encapsulated 5-hexenoic acid ligand species peaks appear significantly broadened and upfield shifted due to the coordination of the species to the negatively charged {$Mo_{132}$} framework, in addition to the confined nature of the ligand environment. The resultant peaks appear at **α'** = 4.5 ppm, **β'/γ'** = 3.8 ppm, **i'** = 1.0 ppm, **ii'** = 0.6 ppm, and **iii'** = 0.8 ppm, with shifts of **α-α'** = --1.4 ppm, **β-β'/γ-γ'** = -1.3 ppm. **i-i'** = -1.4 ppm, **ii-ii'** = -1.1 ppm, and **iii-iii'** = -1.3 ppm.

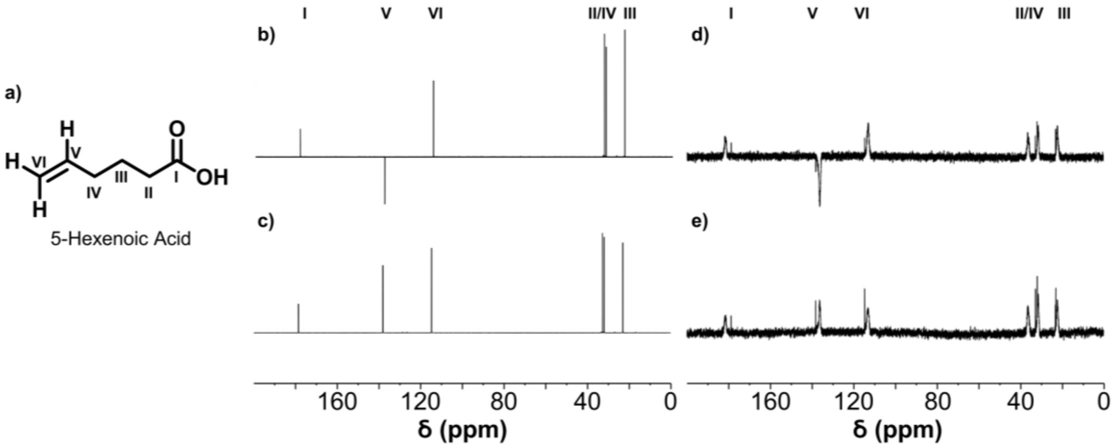


**Figure S41: a)** Assignment of 5-hexenoic acid for use in $^{13}$C NMR spectra labelling, **b)** $^{13}$C DEPTQ NMR spectrum of 5-hexenoic acid in $D_2O$, **c)** $^{13}$C NMR spectrum of 5-hexenoic acid in $D_2O$, **d)** $^{13}$C DEPTQ NMR spectrum of **7** in $D_2O$, and **e)** $^{13}$C NMR spectrum of **7** in $D_2O$. For the $^{13}$C DEPTQ NMR measurements signals arising due to quaternary carbon nuclei ($C_q$) and methylene ($CH_2$) carbon nuclei appear pointing upwards, whilst methine (CH) and methyl ($CH_3$) carbon nuclei appear pointing downwards. The peaks arising from the solvated 5-hexenoic ligand species are highlighted: **I** = 178 ppm (-**C**$OO^-$), **II/IV** = 32 ppm ((-**C**$H_2$)$_2$), **III** = 22 ppm (-**C**$H_2$), **V** = 138 ppm (-**C**H),

and **VI** = 115 ppm (-**C**$H_2$). The encapsulated 5-hexenoic acid ligand species appear slightly shifted and broadened due to the same effects expressed in the $^1$H NMR spectra.

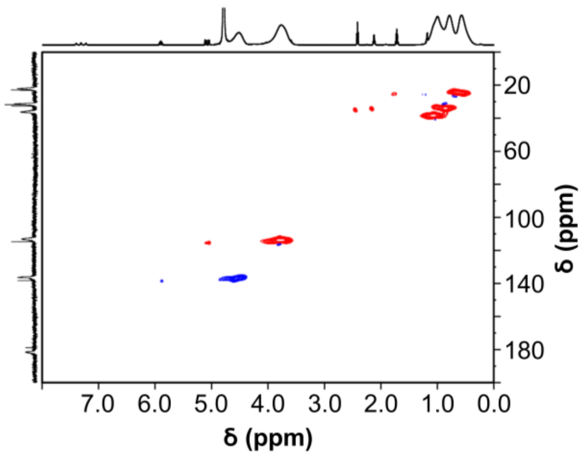


**Figure S42:** $^1$H-$^{13}$C multiplicity-edited HSQC 2D NMR spectrum of **7** in $D_2O$. Here, CH and $CH_3$ signals are highlighted in blue, whilst $CH_2$ signals are highlighted in red. The spectrum confirms the correlation between peaks originating from the free 5-hexenoic acid and the related encapsulated peak signals, in both the $^{13}$C and $^1$H spectra.

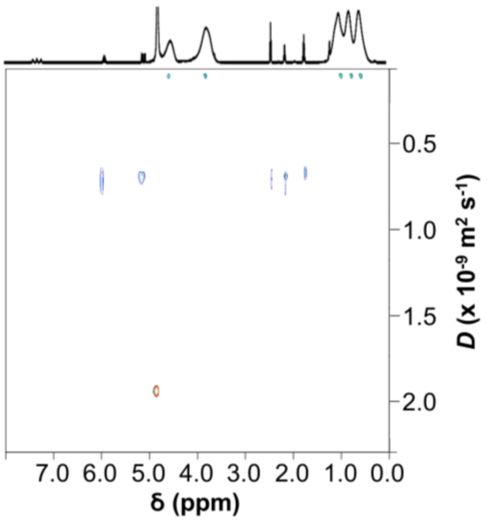


**Figure S43:** $^1$H DOSY NMR spectrum of **7** in $D_2O$. The signals arising from the solvated 5-hexenoic acid ligand species (**αβγi ii iii**) and the encapsulated 5-hexenoic acid ligand species (**α'β'γ'i'ii'iii'**) are highlighted. The solvated 5-hexenoic acid ligands have a diffusion coefficient of approximately 7.02 x $10^{-10}$ $m^2$ $s^{-1}$, whilst the encapsulated 5-hexenoic acid signals possess diffusion coefficients of approximately 1.04 x $10^{-10}$ $m^2$ $s^{-1}$. The large discrimination of the two values provides further evidence of their origins from species in two different domains. An additional peak arising from water has a diffusion coefficient of 1.94 x $10^{-9}$ $m^2$ $s^{-1}$.

**(17) Characterization of 8 – {$Mo_{132}$(Sorbic Acid)}**

*(17.1) Thermogravimetric Analysis of 8*

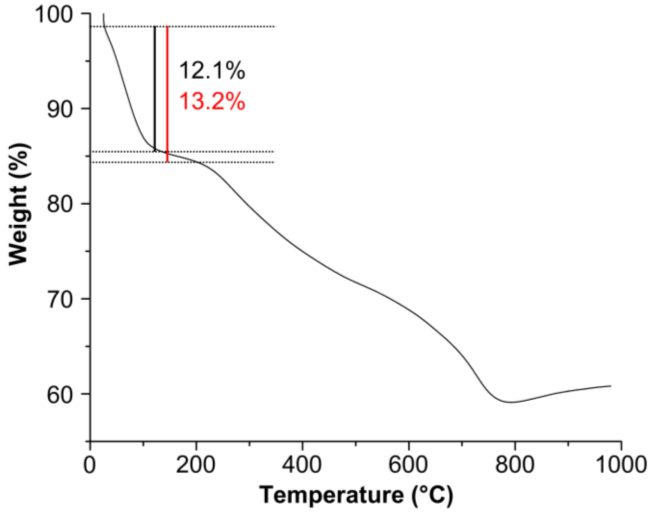


**Figure S44:** TGA of **8**. The percentage weight loss due to water loss between 50-225 °C is highlighted, representing approximately 190 solvent molecules.

*(17.2) NMR Study of 8*

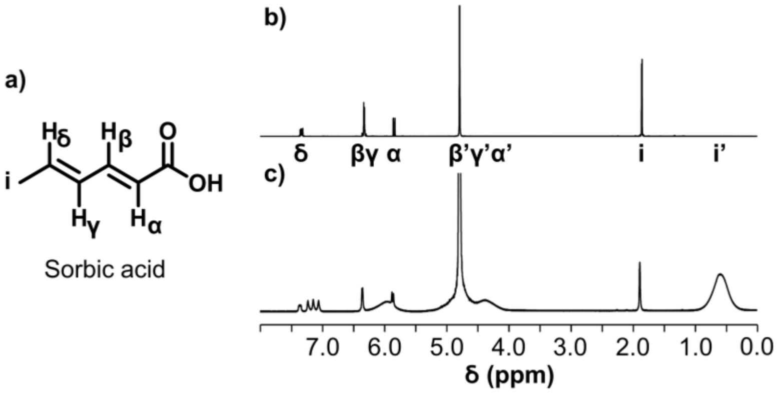


**Figure S45: a)** Structure of sorbic acid with labelled proton groups, **b)** $^1$H NMR spectrum of sorbic acid in $D_2O$, and **c)** $^1$H NMR spectrum of **8** in $D_2O$. The peaks arising from the solvated sorbic acid ligand species are highlighted: **α** = 5.9 ppm, **β**/**γ** = 6.4 ppm, **δ** = 7.4 ppm, and **i** = 1.9 ppm. The encapsulated sorbic acid ligand species peaks appear significantly broadened and upfield shifted due to the coordination of the species to the negatively charged {$Mo_{132}$} framework, in addition to the confined nature of the ligand environment. The resultant peaks appear at **α'** = 4.4 ppm,

**β'/γ'** both signals are obscured by the water peak at approximately 4.8 ppm, **δ'** = 5.9 ppm, and **i'** = 0.6 ppm, with shifts of **α-α'** = -1.5 ppm, **δ-δ'** = -1.6 ppm, and **i-i'** = -1.3 ppm.

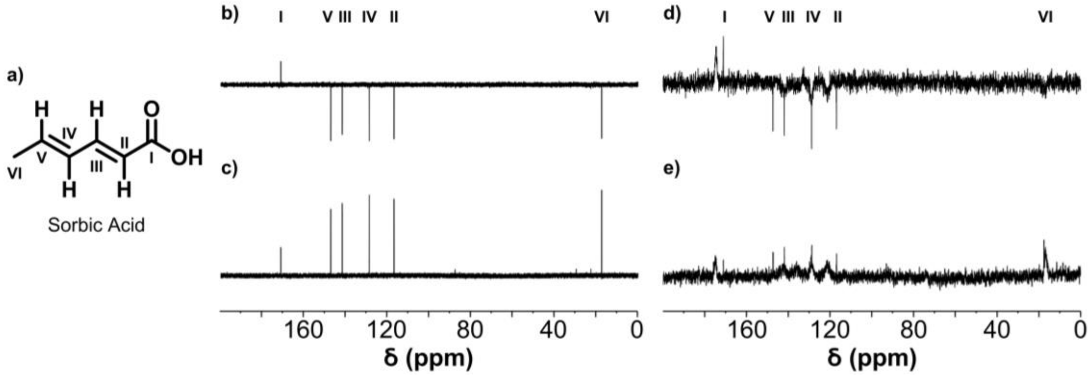


**Figure S46: a)** Assignment of sorbic acid for use in $^{13}C$ NMR spectra labelling, **b)** $^{13}C$ DEPTQ NMR spectrum of sorbic acid in $D_2O$, **c)** $^{13}C$ NMR spectrum of sorbic acid in $D_2O$, **d)** $^{13}C$ DEPTQ NMR spectrum of **8** in $D_2O$, and **e)** $^{13}C$ NMR spectrum of **8** in $D_2O$. For the $^{13}C$ DEPTQ NMR measurements signals arising due to quaternary carbon nuclei ($C_q$) and methylene ($CH_2$) carbon nuclei appear pointing upwards, whilst methine (CH) and methyl ($CH_3$) carbon nuclei appear pointing downwards. The peaks arising from the solvated sorbic ligand species are highlighted: **I** = 171 ppm (-**C**OO$^-$), **II** = 117 ppm (-**C**H), **III** = 142 ppm (-**C**H), **IV** = 129 ppm (-**C**H), **V** = 147 ppm (-**C**H), and **VI** = 18 ppm (-**C**$H_3$). The encapsulated sorbic acid ligand species appear slightly shifted and broadened due to the same effects expressed in the $^1H$ NMR spectra.

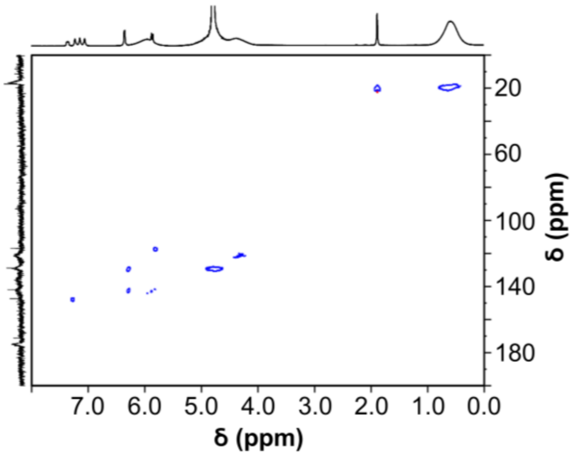


**Figure S47:** $^1H$-$^{13}C$ multiplicity-edited HSQC 2D NMR spectrum of **8** in $D_2O$. Here, CH and $CH_3$ signals are highlighted in blue. The spectrum confirms the correlation between peaks originating from the free sorbic acid and the related encapsulated peak signals, in both the $^{13}C$ and $^1H$ spectra.

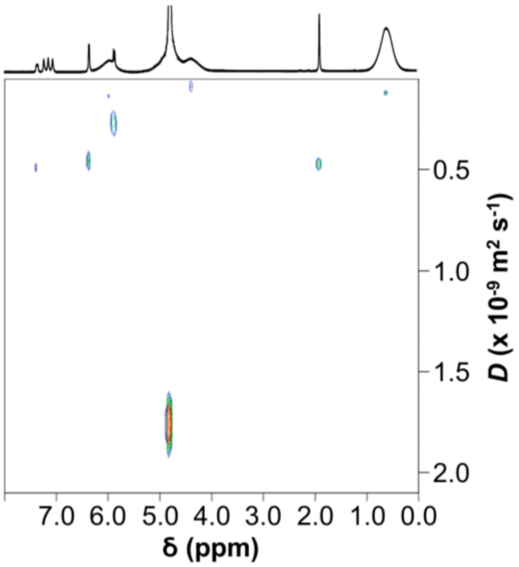


**Figure S48:** $^1$H DOSY NMR spectrum of **8** in $D_2O$. The signals arising from the solvated sorbic acid ligand species (**αβγδi**) and the encapsulated sorbic acid ligand species (**α'β'γ'δ'i'**) are highlighted. The solvated sorbic acid ligands have a diffusion coefficient of approximately 4.66 x $10^{-10}$ $m^2$ $s^{-1}$, whilst the encapsulated sorbic acid signals possess diffusion coefficients of approximately 1.08 x $10^{-10}$ $m^2$ $s^{-1}$. The discrimination of the two values provides further evidence of their origins from species in two different domains. An additional peak arising from water has a diffusion coefficient of 1.79 x $10^{-9}$ $m^2$ $s^{-1}$.

## (18) Thiol-containing Guest Uptake

***Investigation of Uptake between Pure {$Mo_{132}(SO_4)_{30}$}, {$Mo_{132}(SO_4)_{30}$} plus acetic acid, and {$Mo_{132}(SO_4)_{30}$} plus alkene ligands, with Thiol-Containing Alkyl Guests***

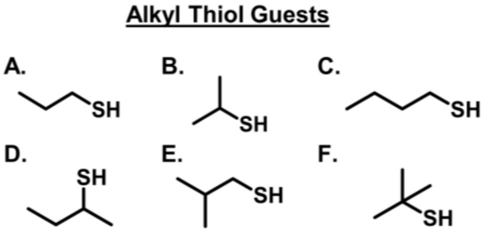


**Figure S49:** Guests used for encapsulation studies: propanethiol (**A**), 2-propanethiol (**B**), 1-butanethiol (**C**), 2-butanethiol (**D**), 2-methyl-1-proanethiol (**E**), and 2-methyl-2-propanethiol.

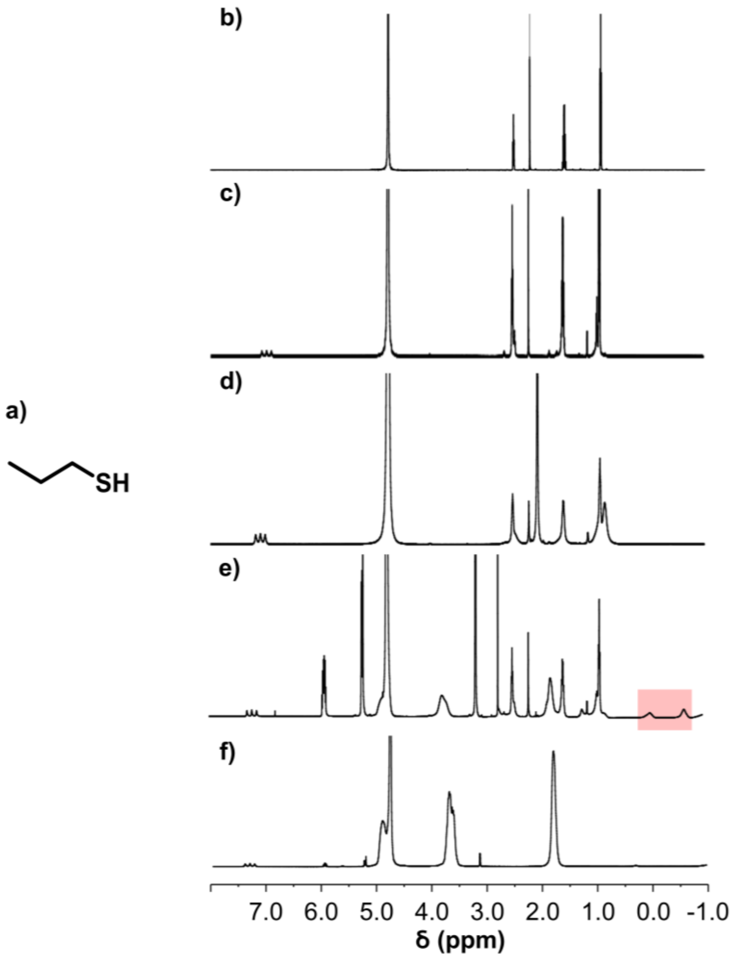


**Figure S50: a)** Structure of 1-propanethiol, and $^{1}H$ NMR spectra of **b)** pure 1-propanethiol, **c)** $\{Mo_{132}(SO_4)_{30}\}$ with added 1-propanethiol, **d)** $\{Mo_{132}(SO_4)_{30}\}$ and OAc (60 equivalents) with added 1-propanethiol, **e) 5** with added 1-propanethiol with a methanesulfonic acid external reference (2.8 ppm), and **f) 5** in $D_2O$. A broad peak related to the encapsulation of the 1-propanethiol guest is highlighted by the red box for **5**.

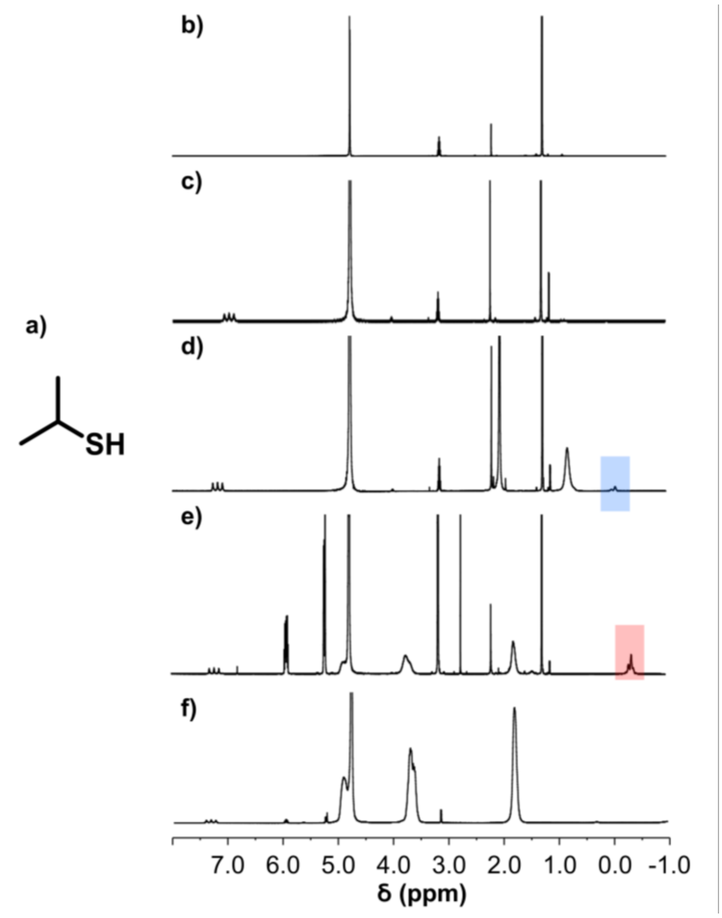


**Figure S51: a)** Structure of 2-propanethiol, and $^{1}H$ NMR spectra of **b)** pure 2-propanethiol, **c)** $\{Mo_{132}(SO_4)_{30}\}$ with added 2-propanethiol, **d)** $\{Mo_{132}(SO_4)_{30}\}$ and OAc (60 equivalents) with added 2-propanethiol, **e) 5** with added 2-propanethiol with a methanesulfonic acid external reference (2.8 ppm), and **f) 5** in $D_2O$. A broad peak related to the encapsulation of the 2-propanethiol guest is highlighted by the red box for **5** and a blue box for $\{Mo_{132}(OAc)_{30}\}$.

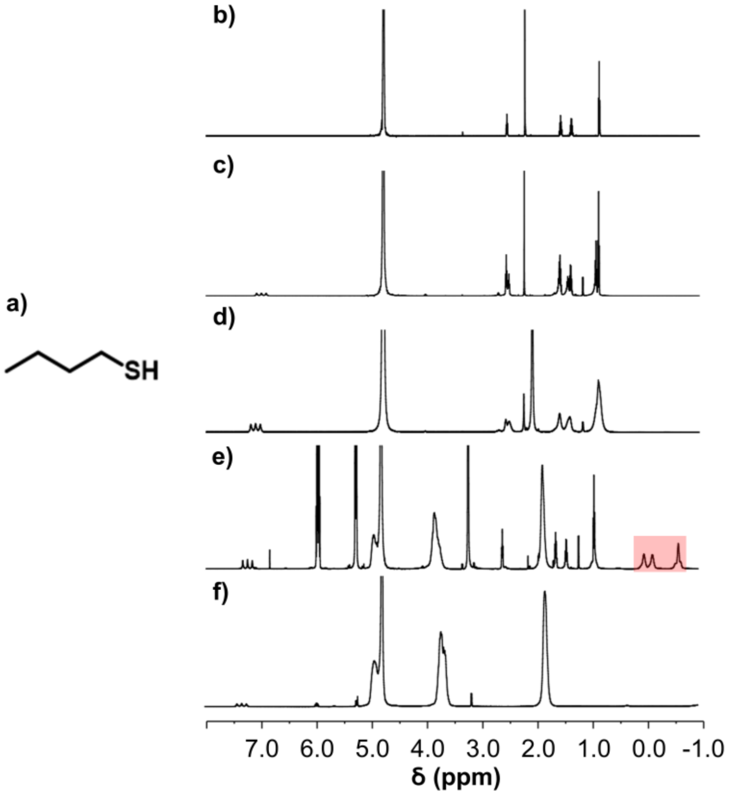


**Figure S52: a)** Structure of 1-butanethiol, and $^{1}$H NMR spectra of **b)** pure 1-butanethiol, **c)** $\{Mo_{132}(SO_4)_{30}\}$ with added 1-butanethiol, **d)** $\{Mo_{132}(SO_4)_{30}\}$ and OAc (60 equivalents) with added 1-butanethiol, **e) 5** with added 1-butanethiol with a methanesulfonic acid external reference (2.8 ppm), and **f) 5** in $D_2O$. A broad peak related to the encapsulation of the 1-butanethiol guest is highlighted by the red box for **5**.

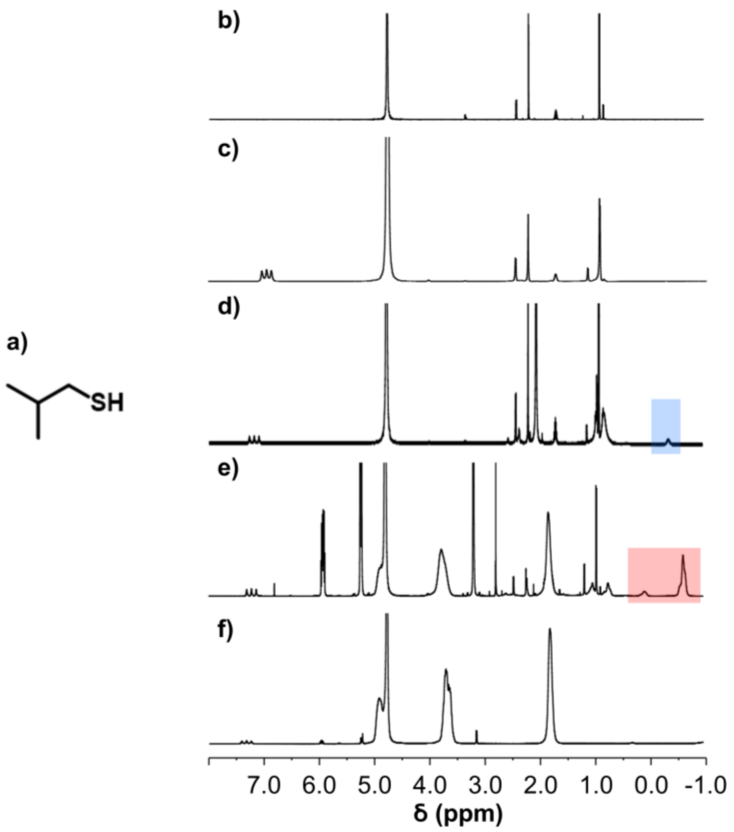


**Figure S53: a)** Structure of 2-methyl-1-propanethiol, and $^1H$ NMR spectra of **b)** pure 2-methyl-1-propanethiol, **c)** $\{Mo_{132}(SO_4)_{30}\}$ with added 2-methyl-1-propanethiol, **d)** $\{Mo_{132}(SO_4)_{30}\}$ and OAc (60 equivalents) with added 2-methyl-1-propanethiol, **e) 5** with added 2-methyl-1-propanethiol with a methanesulfonic acid external reference (2.8 ppm), and **f) 5** in $D_2O$. A broad peak related to the encapsulation of the 2-methyl-1-propanethiol guest is highlighted by the red box for **5** and a blue box for $\{Mo_{132}(OAc)_{30}\}$.

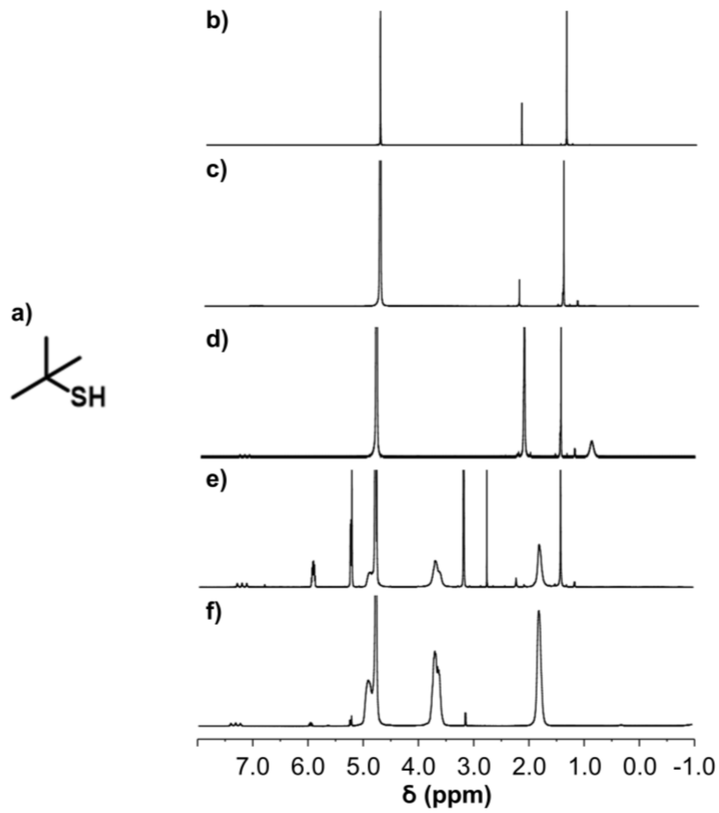


**Figure S54: a)** Structure of 2-methyl-2-propanethiol, and $^1$H NMR spectra of **b)** pure 2-methyl-2-propanethiol, **c)** $\{Mo_{132}(SO_4)_{30}\}$ with added 2-methyl-2-propanethiol, **d)** $\{Mo_{132}(SO_4)_{30}\}$ and OAc (60 equivalents) with added 2-methyl-2-propanethiol, **e) 5** with added 2-methyl-2-propanethiol with a methanesulfonic acid external reference (2.8 ppm), and **f) 5** in $D_2O$.

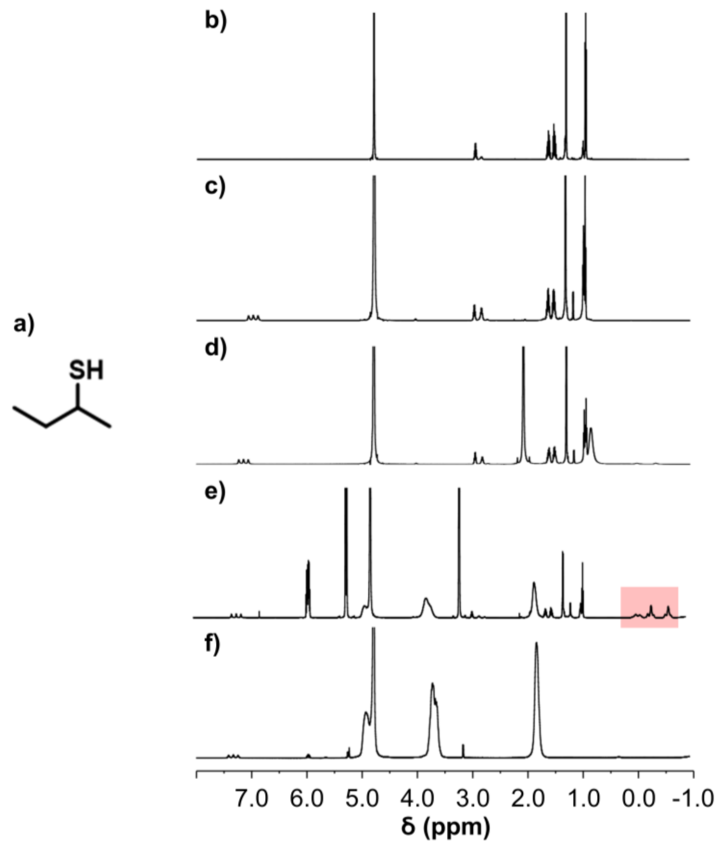


**Figure S55: a)** Structure of 2-butanethiol, and $^{1}$H NMR spectra of **b)** pure 2-butanethiol, **c)** {$Mo_{132}(SO_4)_{30}$} with added 2-butanethiol, **d)** {$Mo_{132}(SO_4)_{30}$} and OAc (60 equivalents) with added 2-butanethiol, **e) 5** with added 2-butanethiol with a methanesulfonic acid external reference (2.8 ppm), and **f) 5** in $D_2O$. A broad peak related to the encapsulation of the 2-butanethiol guest is highlighted by the red box for **2**.

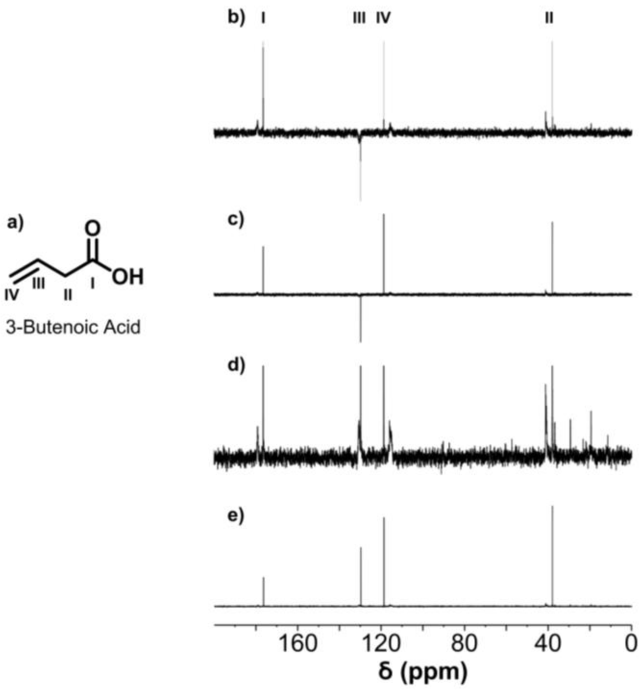


**Figure S56: a)** Assignment of 3-butenoic acid for use in $^{13}C$ NMR spectra labelling, **b)** enhanced $^{13}C$ DEPTQ NMR spectrum of $\{Mo_{132}(SO_4)_{30}\}$ plus 3-butenioic acid and 1-butanethiol in $D_2O$, **c)** $^{13}C$ DEPTQ NMR spectrum of $\{Mo_{132}(SO_4)_{30}\}$ plus 3-butenioic acid and 1-butanethiol in $D_2O$, **d)** enhanced $^{13}C$ NMR spectrum of $\{Mo_{132}(SO_4)_{30}\}$ plus 3-butenioic acid and 1-butanethiol in $D_2O$, and **e**) $^{13}C$ NMR spectrum of $\{Mo_{132}(SO_4)_{30}\}$ plus 3-butenioic acid and 1-butanethiol in $D_2O$. For the $^{13}C$ DEPTQ NMR measurements signals arising due to quaternary carbon nuclei ($C_q$) and methylene ($CH_2$) carbon nuclei appear pointing upwards, whilst methine (CH) and methyl ($CH_3$) carbon nuclei appear pointing downwards. The peaks arising from the solvated 3-butenoic acid ligand species are highlighted: **I** = 179 ppm (-**C**$OO^-$), **II** = 40 ppm (-**C**H), **III** = 130 ppm (-**C**$H_2$), and **IV** = 114 ppm (-**C**$H_2$). The encapsulated 3-butenoic ligand species appear slightly shifted and broadened due to the same effects expressed in the $^1H$ NMR spectra. Additional peaks related to the 1-butanethiol species are present between 10-30 ppm, as seen in **d)**.

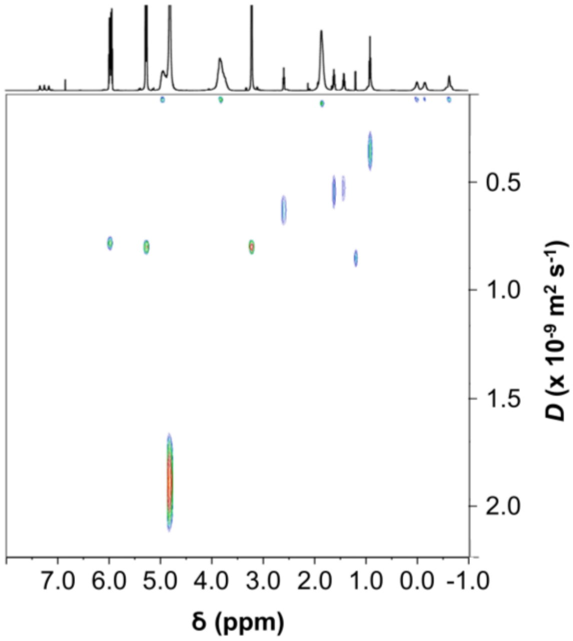


**Figure S57:** $^{1}H$ DOSY NMR spectrum of $\{Mo_{132}(SO_4)_{30}\}$ plus 3-butenoic acid and 1-butanethiol in $D_2O$. The previously assigned solvated 3-butenoic acid ligands have a diffusion coefficient of approximately $7.95 \times 10^{-10}$ m$^2$ s$^{-1}$, whilst the encapsulated 3-butenoic acid signals possess diffusion coefficients of approximately $1.30 \times 10^{-10}$ m$^2$ s$^{-1}$. Further, the peaks assigned to solvated 1-butanethiol possess diffusion coefficients of approximately $5.35 \times 10^{-10}$ m$^2$ s$^{-1}$, whilst those signals assigned to encapsulated 1-butanethiol possess diffusion coefficients of approximately $1.28 \times 10^{-10}$ m$^2$ s$^{-1}$. The close approximation of values for the encapsulated guest species and ligand species indicates that the two separate species are present in a similar environment, the inner cavity of the $\{Mo_{132}\}$ structure. An additional peak arising from water has a diffusion coefficient of $1.88 \times 10^{-9}$ m$^2$ s$^{-1}$.

**Table S6** Number of thiol guests (**A** to **F**) encapsulated within the $\{Mo_{132}(SO_4)_{30}\}$ cavity with added alkene ligands.

| | **Thiol Guest** | 1-Propanethiol | 2-Propanethiol | 1-Butanethiol | 2-Butanethiol | 2-Methyl-1-propanethiol | 2-Methyl-2-propanethiol |
|---|---|---|---|---|---|---|---|
| **Alkene Ligand** | **Label** | **A** | **B** | **C** | **D** | **E** | **F** |
| Acrylic acid | **1** | 27.3 | 8.6 | 24.0 | 20.2 | 21.7 | 2.1 |
| 3-Butenoic acid | **5** | 7.0 | 16.5 | 16.0 | 22.2 | 18.2 | 0.0 |
| 4-Pentenoic acid | **6** | 9.5 | 9.4 | 7.5 | 7.3 | 7.6 | 0.0 |
| 5-Hexenoic acid | **7** | 5.0 | 3.5 | 5.9 | 2.6 | 5.8 | 0.0 |

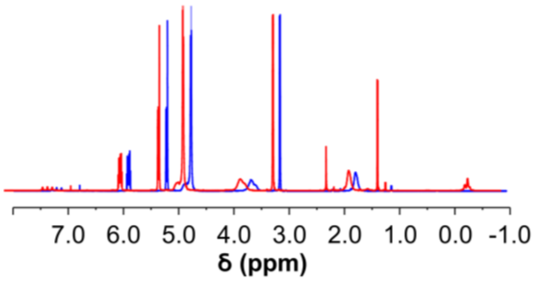


**Figure S58:** $^{1}H$ NMR of $\{Mo_{132}(SO_4)_{30}\}$ plus 3-butenoic acid (blue spectrum), and $\{Mo_{132}(SO_4)_{30}\}$ plus 3-butenoic acid and 1-propanethiol (red spectrum), in $D_2O$, to compare the effects of thiol encapsulation upon alkene encapsulation. The latter spectrum has been shifted downfield slightly to resolve the individual peaks of the two spectra more clearly.

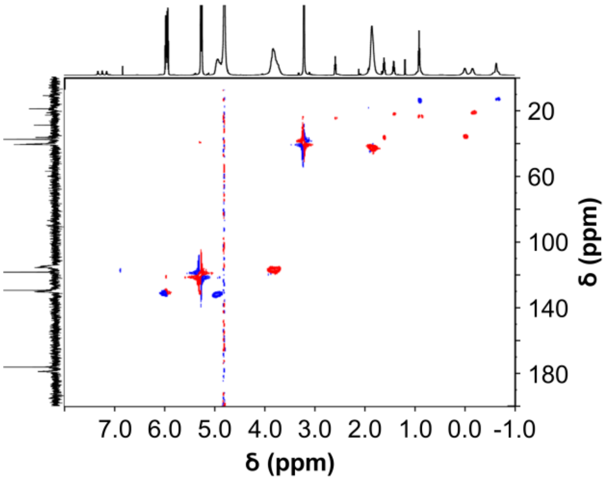


**Figure S59:** $^{1}H$-$^{13}C$ multiplicity-edited HSQC 2D NMR spectrum of $\{Mo_{132}(SO_4)_{30}\}$ plus 3-butenioic acid and 1-butanethiol in $D_2O$. Here, CH and $CH_3$ signals are highlighted in blue, whilst $CH_2$ signals are highlighted in red. The spectrum confirms the previously assigned encapsulated 1-butanethiol peaks in both the $^{13}C$ and $^{1}H$ spectra.

***Investigation of Increasing Temperature on Uptake of 1-butanethiol with $\{Mo_{132}(SO_4)_{30}\}$ plus 3-butenoic acid***

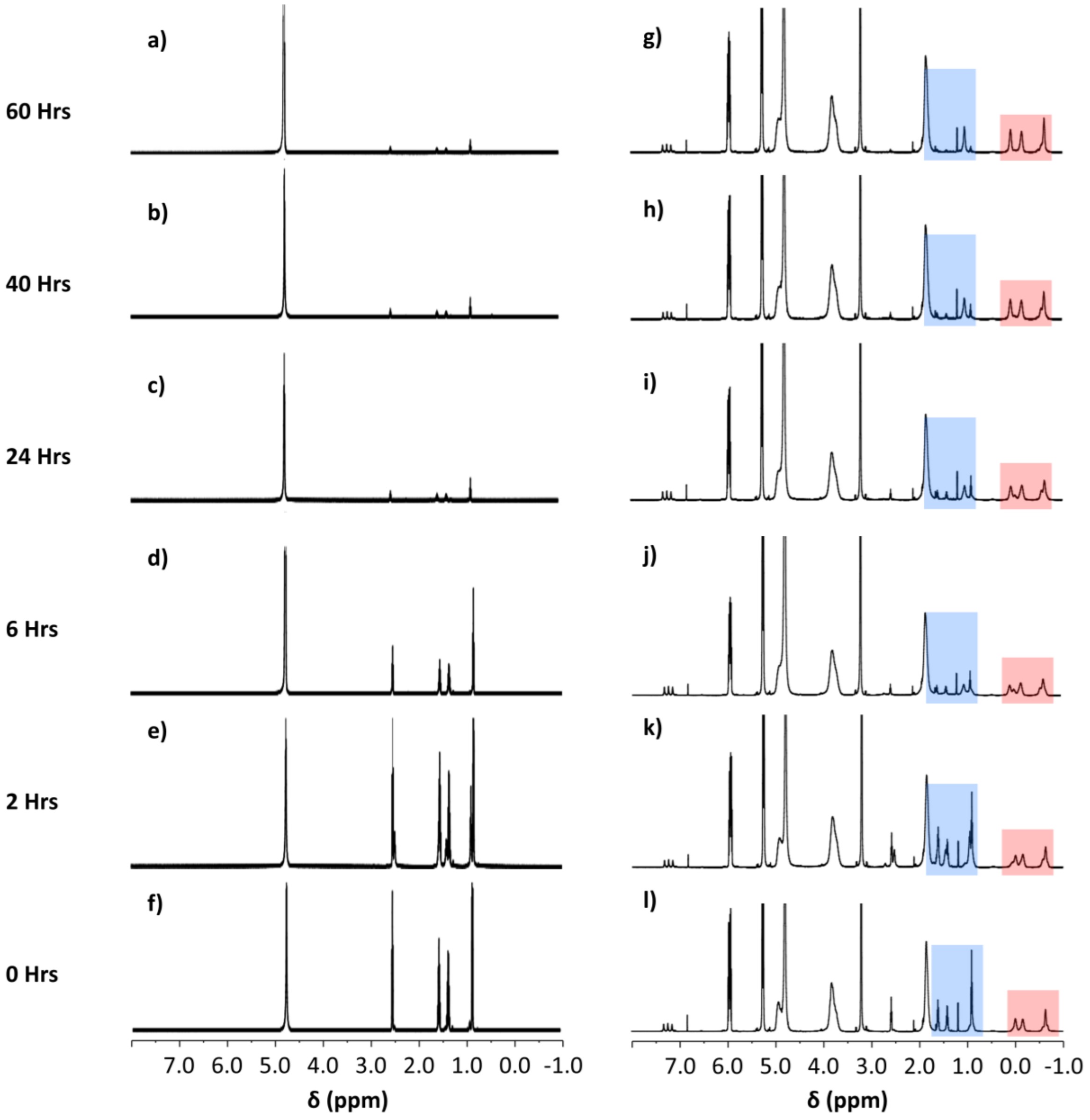


**Figure S60:** $^1$H NMR spectra of 1-butanethiol (**a) – f)**) and 1-butanethiol with $\{Mo_{132}(SO_4)_{30}\}$ and added 3-butenoic acid (**g) – l)**), over a 60-hour period of heating at 70 °C.

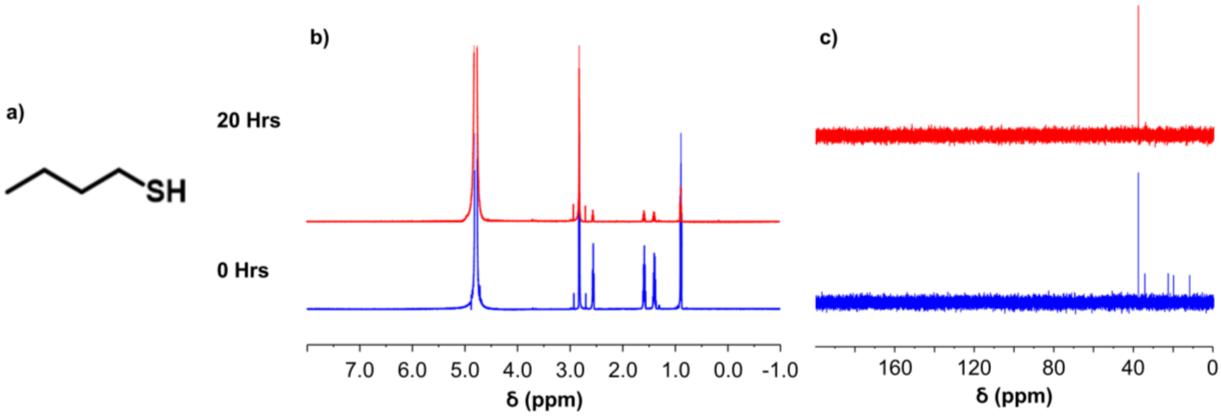


**Figure S61: a)** Structure of 1-butanethiol, **b)** $^1H$ NMR spectra of 1-butanethiol at 300 K prior to and post heating at 343 K for 20 hours, and **c)** $^{13}C$ NMR spectra for the same sample under the same conditions. A clear decrease in peak intensity is observed in both spectra (see peaks in the regions at 1.4-1.6 ppm and 10-38 ppm for the $^1H$ and $^{13}C$ NMR spectra).

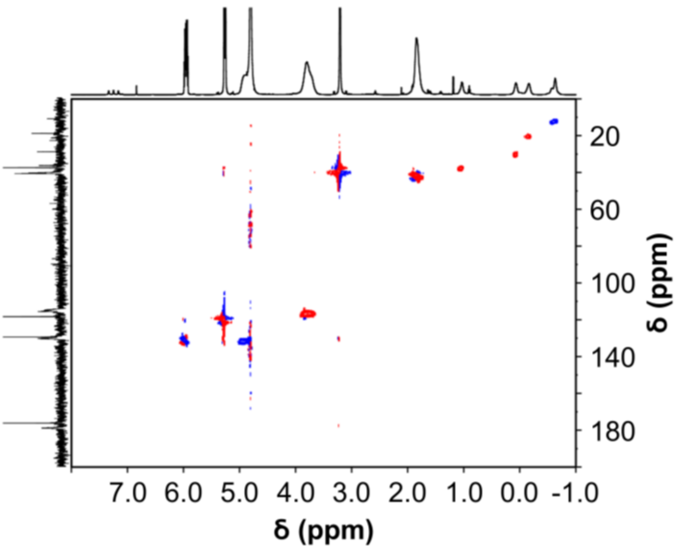


**Figure S62:** $^1H$-$^{13}C$ multiplicity-edited HSQC 2D NMR spectrum of $\{Mo_{132}(SO_4)_{30}\}$ plus 3-butenioic acid and 1-butanethiol in $D_2O$, after heating to 343 K for 60 hours. Here, CH and $CH_3$ signals are highlighted in blue, whilst $CH_2$ signals are highlighted in red. The spectrum confirms the previously assigned encapsulated 1-butanethiol peaks are retained after the heating process has completed, whilst the free thiol species peaks have disappeared.

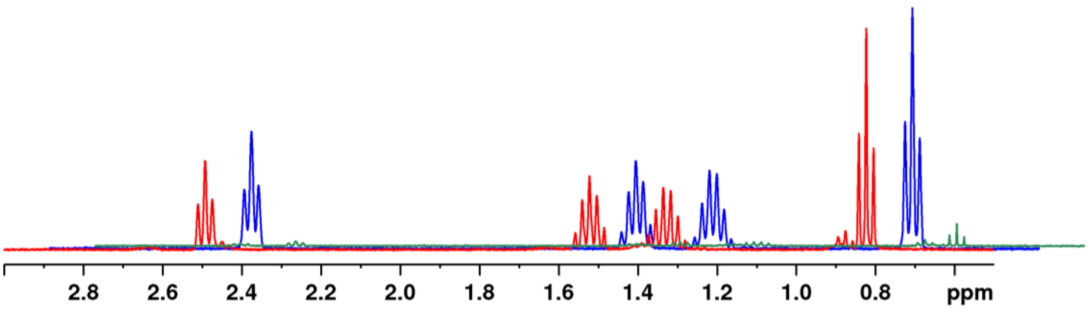


**Figure S63:** $^{1}$H NMR of pure 1-butanethiol in $D_2O$ prior to heating (red spectrum), after heating to 344 K for 40 hours in a screw cap NMR tube (blue spectrum), and after heating to 344 K for a further 20 hours in an uncapped NMR tube (green spectrum). The latter two spectra have been shifted upfield slightly to resolve the individual peaks more clearly.

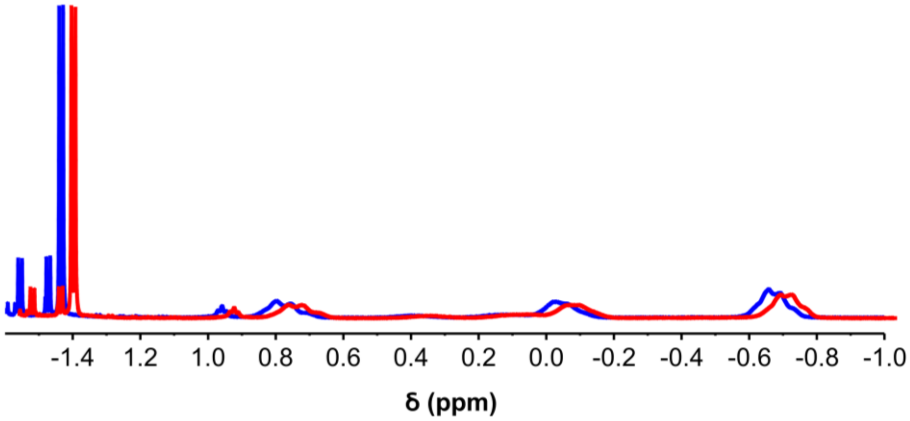


**Figure S64:** $^{1}$H NMR of $\{Mo_{132}(SO_4)_{30}\}$ plus 3-butenoic acid and 1-propanethiol prior to heating (blue spectrum), and $\{Mo_{132}(SO_4)_{30}\}$ plus 3-butenoic acid and 1-propanethiol after heating to 368 K for 60 minutes (red spectrum), in $D_2O$. The latter spectrum has been shifted upfield slightly to resolve the individual peaks of the two spectra more clearly.

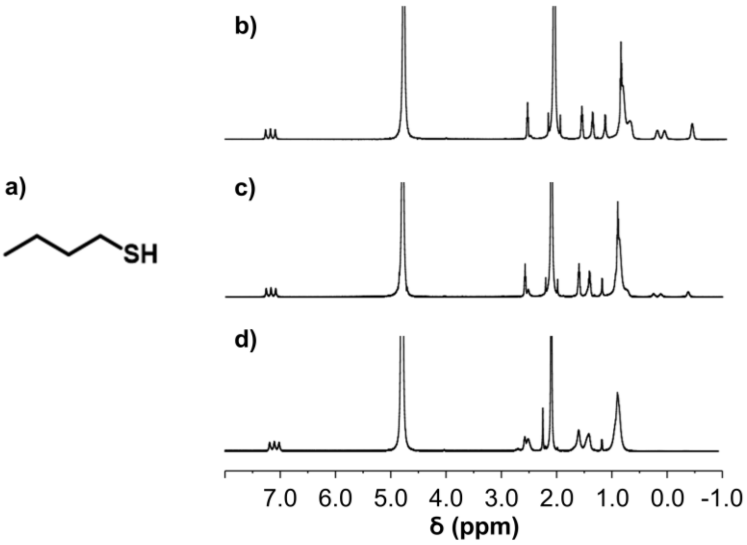


**Figure S65: a)** Structure of 1-butanethiol, and $^{1}H$ NMR spectra of 1-butanethiol plus $\{Mo_{132}(SO_4)_{30}\}$ with added acetate ligands in **b)** 120, **c)** 90, and **d)** 60 equivalents. Peaks arising due to the encapsulation of 1-butanethiol within the central cavity of $\{Mo_{132}\}$ are observed between 0.3 to -0.5 ppm for the spectra with 90 and 120 equivalents of acetate ligands added.

## References


1. A. Müller, E. Krickemeyer, H. Bögge, M. Schmidtmann, and F. Peters, *Angew. Chem., Int. Ed.*, 1998, **37** *(24)*, 3360-3363.

2. S. Floquet, E. Terazzi, A. Hizaji, L. Guénée, C. Piguet, and E. Cadot, *New J. Chem.*, 2012, **36**, 865-868.

3. N. Watfa, S. Floquet, E. Terazzi, W. Salomon, L. Guénée, K. L. Buchwalder, A. Hijazi, D. Naoufal, C. Piguet, and E. Cadot, *Inorganics*, 2015, **3**, 246-266.

4. O. Petina, D. Rehder, E. T. K. Haupt, A. Grego, I. A. Weinstock, A. Merca, H. Bögge, J. Szakács, and A. Müller, *Angew. Chem., Int. Ed.*, 2011, **50**, 410-414.

5. R. Fareghi-Alamdari, N. Zekri, A. J. Moghadam, and M. R. Farsani, *Catalysts Communications*, 2017, **98**, 71-75.

6. A. Müller, S. K. Das, S. Talismanov, S. Roy, E. Beckmann, H. Bögge, M. Schmidtmann, A. Merca, A. Berkle, L. Allouche, Y. Zhou, and L. Zhang, *Angew. Chem., Int. Ed.*, 2003, **42**, 5039-5044.

7. A. Müller, Y. Zhou, H. Bögge, M. Schmidtmann, T. Mitra, E. T. K. Haupt, and A. Berkle, *Angew. Chem., Int. Ed.*, 2006, **45**, 460-465.

8. A. Müller, D. Rehder, E. T. K. Haupt, A. Merca, H. Bögge, M. Schmidtmann, and G. Heinze-Brückner, *Angew. Chem., Int. Ed.*, 2004, **43**, 4466-4470.

9. A. Merca, E. T. K. Haupt, T. Mitra, H. Bögge, D. Rehder, and A. Müller, *Chem. - Eur. J.*, 2007, **13**, 7650-7658.

10. A. Müller, Y. Zhou, L. Zhang, H. Bögge, M. Schmidtmann, M. Dressel, and J. V. Slageren, *Chem. Commun.*, 2004, 2038-2039.

11. L. Zhang, Y. Zhou, and R. Han, *Eur. J. Inorg. Chem.*, 2010, **17**, 2471-2475.

12. A. Müller, L. Toma, H. Bögge, C. Schäffer, and A. Stammler, *Angew. Chem., Int. Ed.*, 2005, **44**, 7757-7761.

13. A. Merca, H. Bögge, M. Schmidtmann, Y. Zhou, E. T. K. Haupt, M. K. Sarker, C. L. Hill, and A. Müller, *Chem. Commun.*, 2008, **8**, 948-950.

14. A. Müller, E. Krickemeyer, H. Bögge, M. Schmidtmann, B. Botar, and M. O. Talismanova, *Angew. Chem., Int. Ed.*, 2003, **42**, 2085-2090.

15. T. Mitra, P. Miró, A.-R. Tomsa, A. Merca, H. Bögge, J. B. Ávalos, J. M. Poblet, C. Bo, and A. Müller, *Chem. - Eur. J.* 2009, **15**, 1844-1852.

16. S. Garai, E. T. K. Haupt, H. Bögge, A. Merca, and A. Müller, *Angew. Chem.*, 2012, **124**, 10680-10683.

17. A. Müller, L. Toma, H. Bögge, M. Henry, E. T. K. Haupt, A. Mix, and F. L. Sousa, *Chem. Commun.*, 2006, 3396-3398.

18. C. Schäffer, A. M. Todea, H. Bögge, O. A. Petina, D. Rehder, E. T. K. Haupt, and A. Müller, *Chem. - Eur. J.*, 2011, **17**, 9634-9639.

19. C. Schäffer, H. Bögge, A. Merca, I. A. Weinstock, D. Rehder, E. T. K. Haupt, and A. Müller, *Angew. Chem., Int. Ed.*, 2009, **48**, 8051-8056.

20. A. Ziv, A. Grego, S. Kopilevich, L. Zeiri, P. Miro, C. Bo, A. Müller, and I. A. Weinstock, *J. Am. Chem. Soc.*, 2009, **131**, 6380-6382.

21. M. Awada, S. Floquet, J. Marrot, M. Haouas, S. P. Morcillo, C. Bour, V. Gandon, V. Coeffard, C. Greck, and E. Cadot, *J. Clust. Sci.*, 2017, **28**, 799-812.

22. T.-L. Lai, M. Awada, S. Floquet, C. Roch-Marchal, N. Watfa, J. Marrot, M. Haouas, F. Taulelle, and E. Cadot, . *Chem. - Eur. J.*, 2015, **21**, 13311-13320.

23. G. M. Sheldrick, *Acta Crystallogr. Sect. A Found. Crystallogr.*, 1990, **46 (6)**, 467.

24. R. Herbst-Irmer, and G. M. Sheldrick, *Acta Crystallogr. Sect. B. Struct. Sci.* 1998, **54 (4)**, 443.

25. L. J. Farrugia, *J. Appl. Crystallogr.*, 1999, **32 (4)**, 837.

26. A. Müller, E. Krickemeyer, H. Bögge, M. Schmidtmann, B. Botar, and M. O. Talismanova, *Angew. Chem., Int. Ed.*, 2003, **42**, 2085-2090.

27. F. M. Wampler, *Plant/Oper. Prog.*, 1988, **7**, 183-189.
